# The potential impact of novel tuberculosis vaccine introduction on economic growth in low- and middle-income countries: A modeling study

**DOI:** 10.1371/journal.pmed.1004252

**Published:** 2023-07-11

**Authors:** Allison Portnoy, Jean-Louis Arcand, Rebecca A. Clark, Chathika K. Weerasuriya, Christinah Mukandavire, Roel Bakker, Edith Patouillard, Nebiat Gebreselassie, Matteo Zignol, Mark Jit, Richard G. White, Nicolas A. Menzies

**Affiliations:** 1 Department of Global Health, Boston University School of Public Health, Boston, Massachusetts, United States of America; 2 Center for Health Decision Science, Harvard T.H. Chan School of Public Health, Boston, Massachusetts, United States of America; 3 Department of International Economics, The Graduate Institute of International and Development Studies, Geneva, Switzerland; 4 Fondation pour les études et recherches sur le développement international (FERDI), Clermont-Ferrand, France; 5 Global Development Network, New Delhi, India; 6 Université Mohammed VI Polytechnique, Rabat, Morocco; 7 TB Modelling Group, London School of Hygiene and Tropical Medicine, London, United Kingdom; 8 Centre for the Mathematical Modelling of Infectious Diseases, London School of Hygiene and Tropical Medicine, London, United Kingdom; 9 Department of Infectious Disease Epidemiology, London School of Hygiene and Tropical Medicine, London, United Kingdom; 10 Coalition for Epidemic Preparedness Innovations, London, United Kingdom; 11 KNCV Tuberculosis Foundation, The Hague, the Netherlands; 12 Department of Health Systems Governance and Financing, World Health Organization, Geneva, Switzerland; 13 Global TB Programme, World Health Organization, Geneva, Switzerland; 14 School of Public Health, University of Hong Kong, Hong Kong SAR, China; 15 Department of Global Health and Population, Harvard T.H. Chan School of Public Health, Boston, Massachusetts, United States of America

## Abstract

**Background:**

Most individuals developing tuberculosis (TB) are working age adults living in low- and middle-income countries (LMICs). The resulting disability and death impact economic productivity and burden health systems. New TB vaccine products may reduce this burden. In this study, we estimated the impact of introducing novel TB vaccines on gross domestic product (GDP) growth in 105 LMICs.

**Methods and findings:**

We adapted an existing macroeconomic model to simulate country-level GDP trends between 2020 and 2080, comparing scenarios for introduction of hypothetical infant and adolescent/adult vaccines to a no-new-vaccine counterfactual. We parameterized each scenario using estimates of TB-related mortality, morbidity, and healthcare spending from linked epidemiological and costing models. We assumed vaccines would be introduced between 2028 and 2047 and estimated incremental changes in GDP within each country from introduction to 2080, in 2020 US dollars. We tested the robustness of results to alternative analytic specifications. Both vaccine scenarios produced greater cumulative GDP in the modeled countries over the study period, equivalent to $1.6 (95% uncertainty interval: $0.8, 3.0) trillion for the adolescent/adult vaccine and $0.2 ($0.1, 0.4) trillion for the infant vaccine. These GDP gains were substantially lagged relative to the time of vaccine introduction, particularly for the infant vaccine. GDP gains resulting from vaccine introduction were concentrated in countries with higher current TB incidence and earlier vaccine introduction. Results were sensitive to secular trends in GDP growth but relatively robust to other analytic assumptions. Uncertain projections of GDP could alter these projections and affect the conclusions drawn by this analysis.

**Conclusions:**

Under a range of assumptions, introducing novel TB vaccines would increase economic growth in LMICs.

## Author summary

**Why was this study done?:**

- Most individuals who develop tuberculosis (TB) are working age adults living in low- and middle-income countries (LMICs), and the resulting disability and death impact economic productivity and burden health systems, but new TB vaccine candidates may reduce this burden.
- Earlier studies have estimated the impact of individual diseases and health risks on rates of economic growth, but the potential gains to economic growth that could be produced by introducing novel TB vaccines have not been previously estimated.

**What did the researchers do and find?:**

- In this study, we estimated the impact of introducing novel TB vaccines on gross domestic product (GDP) growth in 105 LMICs, using a macroeconomic model to simulate country-level GDP trends between 2020 and 2080, in 2020 US dollars.
- We compared scenarios for introduction of infant and adolescent/adult vaccine candidates to a no-new-vaccine counterfactual.
- Compared to the no-new-vaccine counterfactual, both vaccination scenarios produced greater cumulative GDP in the modeled countries over the study period, equivalent to $1.6 (95% uncertainty interval: $0.8, 3.0) trillion for the adolescent/adult vaccine and $0.2 ($0.1, 0.4) trillion for the infant vaccine.
- These GDP gains were substantially lagged relative to the time of vaccine introduction and concentrated in countries with higher current TB incidence.

**What do these findings mean?:**

- This study demonstrates that introducing new TB vaccines could increase economic growth in LMICs.
- The results of this research could inform decision-making around how new TB vaccines are developed and introduced.

## Background

In 2021, an estimated 10.6 million individuals fell ill with tuberculosis (TB), and 1.5 million individuals died from TB [1]. Developing new safe, affordable, and effective TB vaccines is seen as a necessary step for more rapidly reducing disease incidence and mortality, and their successful development is a central component of the End TB Strategy approved by the World Health Assembly in 2014 [2,3]. While promising vaccine candidates exist, substantial additional resources will be needed to further develop these candidates. To judge whether these investments are justified, it is important to understand the full range and magnitude of benefits that could result from new TB vaccines and from different perspectives on what constitutes value.

The primary approach for judging the value of health technologies has been through quantifying the additional health that is produced through introducing the technologies. These health benefits can be denominated in disease-specific measures such as the number of infections averted, or through generic measures such as the number of disability-adjusted life years (DALYs) averted, a measure that combines improvements in the length and quality of life and which is used to make comparisons across diseases. A number of studies have assessed the possible health benefits of TB vaccines [4,5], projecting potentially large health benefits for TB vaccine introduction in high-incidence settings. Studies considering the global impact of TB vaccine reduction have estimated up to 40 to 50 million TB cases could be averted by an effective vaccine by 2050 [6,7], as well as substantial reductions in TB drug resistance [8]. Other studies have placed a monetary value on the health benefits generated by TB vaccine introduction, either by reference to the opportunity cost of healthcare spending [9] or individual willingness-to-pay to reduce health risks [10]. In addition to these health impacts, studies have quantified the consequences of TB vaccines in terms of reduced income losses due to averted TB [7,11] and reductions in catastrophic health expenditures for TB-affected households [11].

In addition to these individual-level outcomes, it is possible that TB vaccine introduction could have consequences at the level of the whole economy. In contrast to most other vaccine-preventable diseases, most individuals who develop TB are working age adults, with 81% of all notified TB cases in 2021 occurring among individuals 15 to 64 years old [12]. While TB is less prevalent than some other infectious diseases, individuals who develop TB disease experience a long period of illness during which they may not be able to work, followed by a course of treatment that can take 6 to 24 months to complete. Nationally representative surveys conducted among TB patients have described substantial income losses due to reduced ability to work, both before a diagnosis is made and during the treatment episode [1]. For individuals surviving TB, ongoing chronic disability can affect productivity and ability to afford basic needs [13,14]. Moreover, over 10% of individuals with TB die from the disease, and both these fatal and nonfatal effects will have consequences for labor force size and participation, which could impact rates of economic growth within TB-affected countries.

Earlier studies have estimated the impact of individual diseases and health risks on rates of economic growth [15–18]. However, the potential gains to economic growth that could be produced by introducing novel TB vaccines have not been previously estimated. In this study, we examined the impact of introducing novel TB vaccines on gross domestic product (GDP) and GDP growth in 105 low- and middle-income countries (LMICs) and evaluated how these impacts varied over time and according to different country characteristics.

## Methods

### Analytic approach

We used a mathematical model of *Mycobacterium tuberculosis* (*Mtb*) transmission, progression, care, and prevention to simulate changes in population health outcomes that would be produced by introduction of a new TB vaccine as compared to a “no-new-vaccine” counterfactual. In this counterfactual, we assumed that TB trends would follow their historical trajectory, consistent with ongoing provision of TB treatment and prevention services at current quality and coverage levels, including provision of neonatal BCG vaccination to prevent severe disease in infants. We simulated these scenarios in each of 105 LMICs and estimated economic outcomes using a related costing model that simulated changes in health service costs (TB vaccination, TB diagnosis and treatment, HIV treatment) affected by vaccine introduction. Detailed methods and outcomes of these models are described elsewhere [6,9] and are summarized in the Supporting information (Exhibit A in S1 Appendix). We used the results of these analyses to generate inputs for an existing macroeconomic model, which we used to translate changes in disease-related mortality, morbidity, and health service utilization into projected macroeconomic outcomes. This study is reported as per the Consolidated Health Economic Evaluation Reporting Standards 2022 (CHEERS 2022) statement (Exhibit B in S1 Appendix) [19]. Any changes to the analysis that were required are also described; no prospective analysis plan was developed.

### Analytic scenarios

We simulated the macroeconomic impact of TB vaccine introduction for 2 separate vaccine products, both assumed to prevent disease with a 10-year average duration of protection. These characteristics were based on World Health Organization (WHO) Preferred Product Characteristics (PPCs) for novel TB vaccines [20]. The infant TB vaccine was assumed to be effective only among individuals without prior *Mtb* infection with 80% efficacy and would be introduced as part of the routine infant vaccine schedule in each country. The adolescent/adult TB vaccine was assumed to be effective for both infected and uninfected individuals with 50% efficacy and would be provided through an initial mass vaccination campaign of all age groups as well as routine vaccination of 9-year-olds. We examined scenarios for each vaccine product individually and assumed that vaccination would be introduced and scaled up over a 5-year period. The vaccine introduction year varied by country (based on an analysis of factors affecting new vaccine adoption), ranging from 2028 and 2047 [6]. We compared these scenarios to the counterfactual “no-new-vaccine” scenario in order to calculate the incremental impact of vaccine introduction on country GDP.

For the costs of the vaccine program for a hypothetical novel vaccine product, we assumed a proxy vaccine price based on the price paid by Gavi, the Vaccine Alliance, for human papillomavirus (HPV) vaccine ($4.60) along with an injection supply cost per dose of $0.11 and a 5% wastage rate [21,22]. In the first year of the vaccine program, we also assumed a one-time vaccine introduction cost of $0.65 and $2.40 per targeted individual for infant and adolescent/adult vaccines, respectively [23], and vaccine delivery costs based on a meta-analysis of childhood [24] and HPV vaccine delivery unit costs for the infant and adolescent/adult vaccines, respectively. In the base–case analysis, in addition to vaccination program costs, we included costs from the societal perspective for direct medical costs of TB/HIV treatment (both government- and patient-level costs), and patient-level direct nonmedical costs (additional details on cost assumptions are described elsewhere [9]). For all unit cost inputs related to service provision, we subtracted the share contributed by international donors to obtain unit costs reflecting the contribution of domestic resources to service provision. For vaccination costs, we assumed a donor share stratified by World Bank income level (69.4% for low-income countries, 39.4% for lower-middle-income countries, and 0.2% for upper-middle-income countries) based on Ikilezi and colleagues [25]. For TB diagnosis and treatment costs, we estimated country-specific donor shares using spending estimates from the WHO Global TB Report 2022 (Exhibit C in S1 Appendix) [1]. For antiretroviral therapy costs, we assumed a country-specific donor share based on spending estimates from the Global Burden of Disease Collaborative Network at IHME (Exhibit C in S1 Appendix) [26]. All costs underlying the macroeconomic analysis were calculated in constant 2020 US dollars (Exhibit D in S1 Appendix).

We simulated macroeconomic outcomes over 2028 to 2080 to capture the long-term consequences of vaccine introduction. For the period during and immediately following vaccine introduction (2028 to 2050), we simulated model inputs using the *Mtb* transmission and costing models described earlier. For long-term outcomes (2050 to 2080), we extrapolated population health inputs by applying United Nations Population Division mortality rate projections by age and year to the population of each country at the end of 2050 for each analytic scenario [27]. Cost inputs for 2050 to 2080 were assumed to change in proportion to population growth (based on 2050 values) for each scenario.

### Macroeconomic model

The WHO EPIC (Economic Projections of Illness and Cost) model is based on a standard human capital-augmented Solow model of economic growth and has been used in prior studies to project the macroeconomic impact of various health conditions [28–33]. We adapted this model to estimate the effect of vaccine introduction on the economic output of each modeled country, as produced by averted TB morbidity and mortality and related changes in health service utilization. Full details of model specification are provided in the Supporting information (Exhibit E in S1 Appendix). Changes in TB mortality were assumed to influence macroeconomic outcomes through changes in the labor supply, with the size and age structure of the available workforce (stock of individuals aged 15 to 69, adjusted for labor force participation and labor quality) modified to reflect the additional survival estimated for the TB vaccination scenarios. We assumed that labor force participation rates and labor quality within each single-year age group were the same across vaccine and no-new-vaccine scenarios. To account for changes in nonfatal health outcomes, we subtracted from this labor supply the years lived with disability (YLDs) estimated for the vaccine introduction scenarios as compared to the no-new-vaccine scenario, for each year of age. These YLDs represent the incremental person-time spent in ill health, scaled by the relative disability associated with a given condition [34]. We did not account for any additional losses of productivity within the household to care for sick individuals but varied this in sensitivity analysis. Changes in domestic health spending (total costs of modeled health services net of the international donor share for these services) were subtracted directly from the total stock of physical capital (tangible assets like buildings and machinery). In sensitivity analysis, we examined alternative assumptions for the macroeconomic impact of changes in healthcare spending. Other variables determining macroeconomic outcomes (savings rate [the percentage of disposable income saved rather than spent on consumption], growth rate of total factor productivity [the change in economic growth that occurs due to factors other than changes in the labor force or capital stock (e.g., technological advancement)], growth rate of educational capital [returns to education that increase labor quality], output elasticity of physical capital [the change in the output that results from a change in physical capital], and depreciation rate [the percentage decrease in the monetary value of assets over time] for each country) were derived from published sources (Exhibit E in S1 Appendix) and assumed to be unaffected by vaccine introduction. With these specifications, we used the EPIC model to project total annual GDP for each modeled country over 2020 to 2080.

### Outcomes

We estimated incremental changes in GDP within each country from vaccine introduction until 2080 by comparing each vaccine scenario to the no-new-vaccine counterfactual. We summarized results as absolute and percentage differences in GDP, changes in per-capita GDP, and time trends in the rate of GDP growth within modeled countries. We report results according to major country groupings (global, WHO high-TB burden grouping [35], World Bank income level [36], and WHO region). We also estimated partial rank correlation coefficients (PRCCs) for the percentage change in GDP under vaccination scenarios as a function of selected country characteristics (current TB incidence level, HIV incidence level, country per-capita GDP, year of vaccine introduction) and country-specific macroeconomic variables (mean values for the savings rate, growth rate of total factor productivity, growth rate of educational capital, output elasticity of physical capital, and depreciation rate). These PRCCs quantify the direction and relative strength of the relationships between the economic gains produced by TB vaccination and each of these country characteristics, controlling for the effects of other characteristics, and are robust to nonlinearity in the estimated relationships [37].

### Statistical analysis

Uncertainty intervals (95% coverage, equal-tailed) for the projected gains in GDP were generated via a second-order Monte Carlo simulation. To implement this, we derived 1,000 simulated trajectories of epidemiological and intervention cost outcomes from the *Mtb* transmission and costing models. For country-specific values for the macroeconomic parameters, we generated future values for each country and year using a semiparametric bootstrapping approach. First, we fit random-effects regression models to estimate country-average values for each parameter, based on recent data (2008 to 2019) reported for each country. Any missing values were assumed to be missing-at-random conditional on WHO world region and country income level. Second, we resampled with replacement from the residuals of these regression models and combined these with the country-average values, producing a time series of future values for each parameter and country. We combined all sources of uncertainty to generate 1,000 estimated trajectories of annual GDP for each modeled scenario and country. We took the mean of these 1,000 simulations to produce point estimate results for each outcome of interest and calculated 95% intervals as the 2.5th and 97.5th percentiles of the distribution of results for each outcome of interest.

### Sensitivity analysis

Compared to the base–case assumptions about vaccine effectiveness and uptake, we examined the following alternative scenarios: (1) we examined a scenario assuming lifelong duration of protection conferred by vaccination, as compared to the base–case assumption of 10-year duration of protection; (2) we examined a scenario assuming 75% efficacy conferred by the adolescent/adult vaccine, as compared to the base–case assumption of 50% efficacy; (3) we examined a “low” coverage scenario (75%, 70%, and 50% coverage for routine infant vaccine delivery, routine adolescent vaccine delivery, and campaign adolescent/adult vaccine delivery, respectively), as compared to the base–case coverage targets; (4) we examined a “high” coverage scenario (95%, 90%, and 90% coverage for routine infant vaccine delivery, routine adolescent vaccine delivery, and campaign adolescent/adult vaccine delivery, respectively), as compared to the base–case coverage targets; (5) we examined an accelerated scale-up scenario in which all countries introduced vaccination in 2025 and achieved instantaneous scale-up to the specified coverage targets, as compared to the base–case vaccine delivery assumptions; and (6) we examined a “routine delivery-only” scenario that removed the one-time campaign delivery component of the adolescent/adult base–case scenario.

Compared to the base–case assumptions about future trends in TB incidence in the no-new-vaccine baseline, we also estimated results under an alternative scenario in which TB incidence was assumed to decline rapidly through the scale-up of existing preventive treatment and case detection, meeting the incidence reduction targets for 2035 described in the WHO End TB Strategy without introduction of a new vaccine [2,3,38].

Compared to the base–case cost assumptions (including TB-related health services costs incurred by patients and domestic government, excluding costs borne by international donors), we examined 3 alternative approaches to the specification of health services costs in the macroeconomic analysis: (1) modifying the base–case to exclude patient-incurred costs, under the assumption that estimated changes in household healthcare spending would be offset by matching changes in other consumption; (2) modifying the base–case to exclude costs borne by domestic government, under the assumption that estimated changes in government spending would be offset by matching changes in taxation; and (3) modifying the base–case to include costs borne by international donors, assuming that the absolute value of donor spending in each country would not change between vaccination and no-new-vaccine scenarios, such that domestic governments would absorb all incremental health service costs.

We also examined how results would change under low and high economic growth trajectories. We operationalized the low-growth specification by estimating results from the 20% of simulations for each country that had the lowest average annual growth over the 2020 to 2080 period under the no-new-vaccine scenario. Similarly, the high-growth specification was estimated from the 20% of simulations for each country with the highest average annual growth over 2020 to 2080 period under the no-new-vaccine scenario.

Finally, we estimated results with an alternative approach for incorporating the impact of TB-related morbidity. Compared to the main analysis, in which nonfatal illness was assumed to reduce the labor supply (with sick individuals unable to participate in the workforce), this specification instead quantified these changes through the income losses experienced by TB-affected households due to reduced productivity. These values were based on data from nationally representative TB patient surveys [39]. These income losses were values in monetary units and added to other costs borne by patients.

### Ethics approval and consent to participate

Not applicable.

## Results

### Total economic impact 2028–2080

Across all 105 LMICs considered in this analysis, the projected economic dividend from adolescent/adult vaccine introduction was estimated to be $1,618 (95% uncertainty interval: $764, 2,988) billion over the 2028 to 2080 period (Table 1). These gains in GDP are equivalent to a 0.033% (0.027, 0.039%) increase in total GDP projected for the study period for all modeled countries.

**Table 1:** Gains to GDP due to adolescent/adult tuberculosis vaccines across 2028–2080.

Country grouping	Absolute gains in GDP (billions 2020 US dollars)	Percentage gain in GDP (%)
All countries	1,618 (764, 2,988)	0.0326% (0.0266%, 0.0388%)
High-TB burden^a^	1,587 (756, 2,906)	0.0395% (0.0328%, 0.0467%)
High-TB/HIV burden^a^	1,413 (693, 2,562)	0.0854% (0.0727%, 0.1003%)
High-MDR/RR-TB burden^a^	1,522 (727, 2,777)	0.0383% (0.0315%, 0.0454%)
	Income level^b^	
Low income	53.5 (24.4, 103)	0.0391% (0.0304%, 0.0474%)
Lower middle income	1,422 (681, 2,594)	0.0842% (0.0723%, 0.0985%)
Upper middle income	143 (38.3, 303)	0.0044% (0.0022%, 0.0060%)
	World region	
AFR	386 (205, 677)	0.1166% (0.1008%, 0.1338%)
AMR	−6.66 (−19.6, −0.63)	−0.0022% (−0.0062%, −0.0001%)
EMR	8.12 (−2.16, 21.0)	0.0056% (−0.0020%, 0.0120%)
EUR	1.75 (−9.31, 12.5)	0.0002% (−0.0027%, 0.0018%)
SEAR	981 (459, 1,819)	0.0846% (0.0690%, 0.1024%)
WPR	247 (95.2, 510)	0.0094% (0.0069%, 0.0119%)

Note: All countries include 105 LMICs analyzed.

Note: Values in parentheses represent equal-tailed 95% uncertainty intervals.

^a^High-TB, high-TB/HIV (HIV-associated TB), and high-MDR/RR-TB (multidrug/rifampicin-resistant TB) burden countries as defined by the World Health Organization.

^b^Low income: GNI per capita of $1,085 or less; lower middle income: GNI per capita of $1,086 to $4,225; upper middle income: GNI per capita of $4,256 to $13,205 (World Bank 2021).

AFR, African region; AMR, Region of the Americas; EMR, Eastern Mediterranean region; EUR, European region; GDP, gross domestic product; GNI, gross national income; LMIC, low- and middle-income country; SEAR, Southeast Asian region; WPR, Western Pacific region.

At $207 ($81, 405) billion, the infant vaccine showed smaller, but still substantial, impacts on GDP over the 2028 to 2080 period (Table 2). These gains are equivalent to a 0.004% (0.003, 0.005%) increase in total GDP across all countries projected for the study period. For both vaccine products, average percentage gains in GDP were substantially higher for the subset of countries identified as “high-TB burden” by WHO.

**Table 2:** Gains to GDP due to infant tuberculosis vaccines across 2028–2080.

Country grouping	Absolute gains in GDP (billions 2020 US dollars)	Percentage gain in GDP (%)
All countries	207 (80.6, 405)	0.0041% (0.0029%, 0.0053%)
High-TB burden^a^	220 (92.7, 416)	0.0054% (0.0040%, 0.0068%)
High-TB/HIV burden^a^	190 (82.8, 354)	0.0114% (0.0085%, 0.0147%)
High-MDR/RR-TB burden^a^	212 (89.6, 403)	0.0053% (0.0039%, 0.0067%)
Income level^b^	Income level^b^	
Low income	3.46 (−0.30, 10.0)	0.0023% (−0.0004%, 0.0044%)
Lower middle income	187 (78.8, 358)	0.0110% (0.0081%, 0.0142%)
Upper middle income	16.2 (−1.31, 43.7)	0.0005% (−0.0001%, 0.0008%)
World region	World region	
AFR	60.3 (25.0, 117)	0.0180% (0.0120%, 0.0249%)
AMR	−3.62 (−6.29, −2.33)	−0.0012% (−0.0021%, −0.0007%)
EMR	−2.16 (−5.33, 0.68)	−0.0017% (−0.0045%, 0.0004%)
EUR	−2.92 (−5.95, −1.54)	−0.0007% (−0.0017%, −0.0003%)
SEAR	107 (45.2, 211)	0.0092% (0.0064%, 0.0128%)
WPR	47.8 (15.8, 104)	0.0018% (0.0011%, 0.0025%)

Note: All countries include 105 LMICs analyzed.

Note: Values in parentheses represent equal-tailed 95% uncertainty intervals.

^a^High-TB, high-TB/HIV (HIV-associated TB), and high-MDR/RR-TB (multidrug/rifampicin-resistant TB) burden countries as defined by the World Health Organization.

^b^Low income: GNI per capita of $1,085 or less; lower middle income: GNI per capita of $1,086 to $4,225; upper middle income: GNI per capita of $4,256 to $13,205 (World Bank 2021).

AFR, African region; AMR, Region of the Americas; EMR, Eastern Mediterranean region; EUR, European region; GDP, gross domestic product; GNI, gross national income; LMIC, low- and middle-income country; SEAR, Southeast Asian region; WPR, Western Pacific region.

### Time trends in economic impact

**Fig 1:**
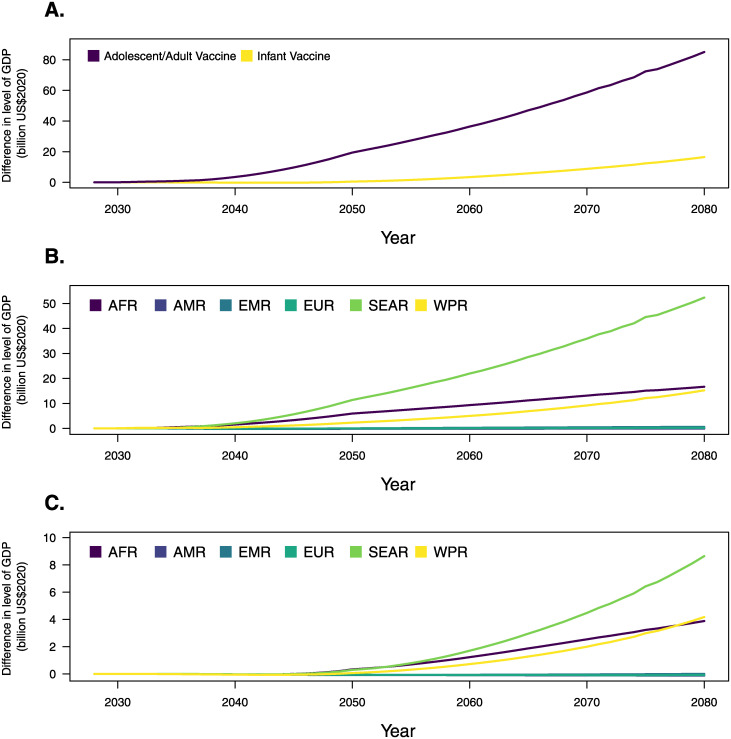
Time trends in gains of GDP (in billions, 2020 US dollars) for all modeled countries, due to adolescent/adult and infant novel tuberculosis vaccination (panel A); due to adolescent/adult novel tuberculosis vaccine, by world region (panel B); due to infant tuberculosis vaccine, by world region (panel C). Note: Panels A, B, and C have different y-axis intervals for readability. AFR, WHO African region; AMR, WHO Region of the Americas; EMR, WHO Eastern Mediterranean region; EUR, WHO European region; GDP, gross domestic product; SEAR, WHO Southeast Asian region; WHO, World Health Organization; WPR, WHO Western Pacific region.

By calendar year, economic benefits were delayed relative to the timing of vaccine introduction, with the greatest economic benefits accruing at the end of the study period (Fig 1). The impact of the adolescent/adult vaccine achieved earlier impacts due to the vaccination of older cohorts where most TB burden lies, whereas the infant vaccine was estimated to produce positive gains over the overall projection period despite only minimal impact prior to 2050.

The WHO regions experiencing the greatest economic growth due to introducing the adolescent/adult and infant novel TB vaccines were the African region (AFR) and Southeast Asian region (SEAR) (Fig 1B and 1C). The Eastern Mediterranean region (EMR) and Western Pacific region (WPR) also achieved positive gains to GDP over 2028 to 2080, whereas the Region of the Americas (AMR) and European region (EUR) with lower TB burden saw positive GDP growth by 2080 but negative gains to GDP over the entire period within the 95% uncertainty intervals. Estimates of the cumulative gains in GDP by decade are given in Exhibits F and G in S1 Appendix.

**Fig 2:**
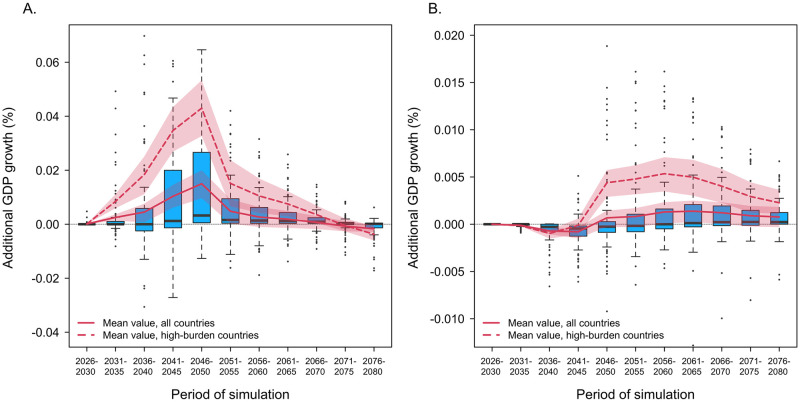
Incremental GDP growth compared to the no-new-vaccine counterfactual in successive 5-year periods from 2026 to 2080 for the adolescent/adult vaccine (panel A) and infant vaccine (panel B). Note: Boxplots represent the distribution of results across individual countries (point estimates), for each 5-year period. Red lines indicate the arithmetic mean of these values for all countries (solid line) and the 30 countries identified as high-TB-burden by WHO (dashed line). Red shaded regions report 95% uncertainty intervals. Note different y-axis scales. GDP, gross domestic product; TB, tuberculosis; WHO, World Health Organization.

Fig 2 displays changes in economic growth produced by each vaccine product for successive 5-year periods until 2080, showing the distribution of individual country-level estimates as well as the average impact across countries. For the adolescent/adult vaccine, the impact of vaccine introduction on the rate of economic growth increased progressively until 2046 to 2050 and then declined over the rest of the analytic period. For the infant vaccine, economic benefits were smaller than estimated for the adolescent/adult vaccine and substantially lagged relative to the timing of vaccine introduction. The average estimate of additional economic growth was negative for the period 2026 to 2045, with the opportunity costs of vaccine spending (e.g., deferred educational investments, reduced saving) outweighing the productivity gains resulting from reduced TB incidence. The economic impact of the infant vaccine scenario was positive for all subsequent periods. For both adolescent/adult and infant vaccine scenarios, incremental changes in GDP growth were substantially higher for the 30 countries identified as high-TB-burden by WHO.

### Relationship between economic impact and country characteristics

**Fig 3:**
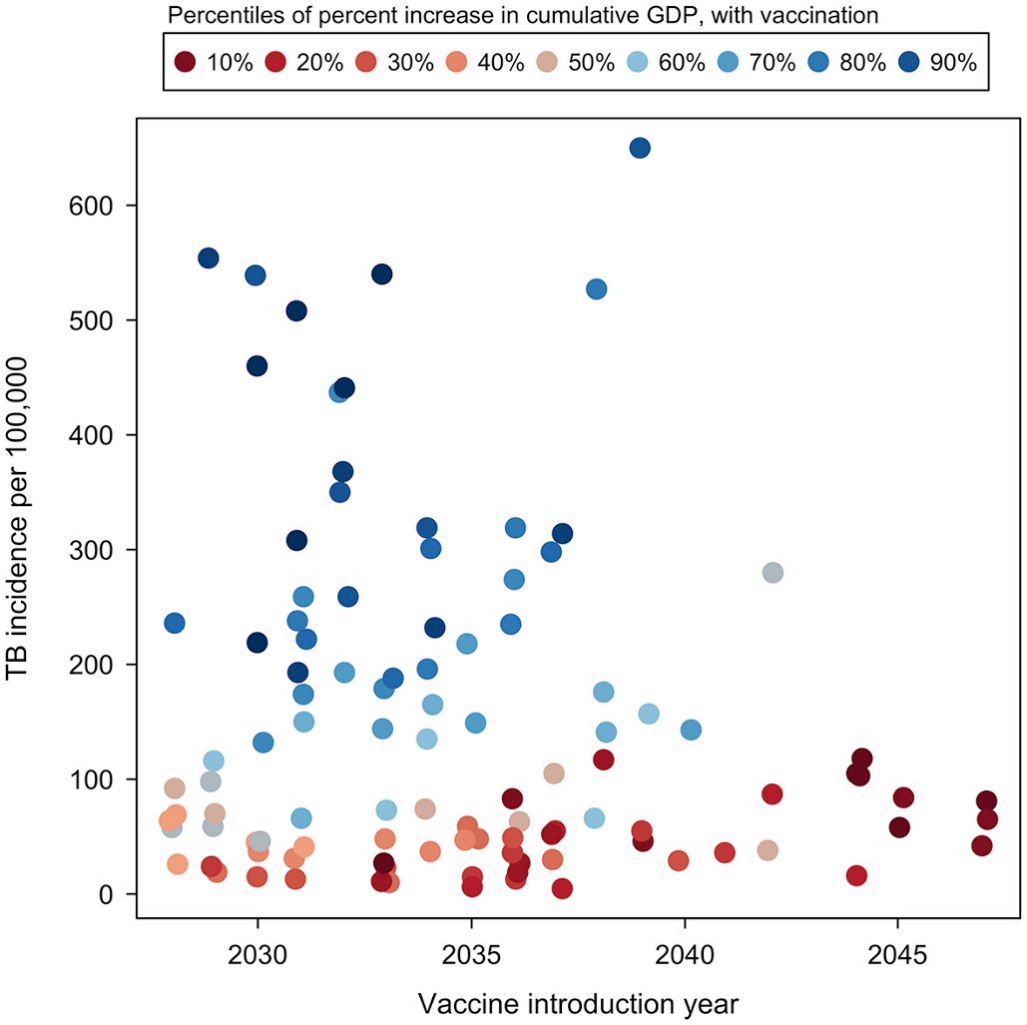
Relationship between additional GDP growth produced by the adolescent/adult vaccine over 2028–2080, current TB incidence level, and vaccine introduction year. Note: Each point represents point-estimate results for an individual country. Color scale ranges from low percentage increase in GDP relative to other countries (reds) to high percentage increase in GDP relative to other countries (blues). Vaccine introduction year values jittered to display overlapping points. GDP, gross domestic product; TB, tuberculosis.

Table 3 reports PRCCs describing the relationship between the percentage gains in GDP produced by each vaccine scenario (percentage increase in GDP over the 2028 to 2080 period) and selected country characteristics. In these analyses, higher current TB incidence was strongly associated with greater economic impact for both vaccine scenarios (*p* < 0.001), with a rank correlation of 0.79 (0.67, 0.91) for the adolescent/adult vaccine and 0.58 (0.42, 0.74) for the infant vaccine, controlling for other factors. Later vaccine introduction year was strongly associated with smaller economic impact (*p* < 0.001), with rank correlations of −0.57 (−0.73, −0.41) for the adolescent/adult vaccine and −0.46 (−0.63, −0.29) for the infant vaccine. Fig 3 shows the relationship between the additional economic growth produced by the adolescent/adult vaccine and current TB incidence rate and vaccine introduction year. Estimated gains in GDP resulting from vaccine introduction were concentrated in countries with high TB incidence that were projected to introduce vaccination early in the study period, with cumulative estimated gains negligible or negative for countries with TB incidence below 125 per 100,000 for the adolescent/adult vaccine. Of the macroeconomic variables, the output elasticity of physical capital had a negative relationship with GDP growth for both vaccine products (*p* = 0.01), and the savings rate had a positive relationship with GDP growth for the infant vaccine (*p* = 0.01), controlling for other predictors (Table 3).

**Table 3:** PRCCs quantifying the relationship between additional GDP growth in each vaccine scenario (relative to the “no-new-vaccine” counterfactual) and selected country characteristics.

	Adolescent/adult vaccine		Infant vaccine	
	Coefficient (95% interval)	*p*-value	Coefficient (95% interval)	*p*-value
TB incidence rate per 100,000	0.79 (0.67, 0.91)	<0.001	0.58 (0.42, 0.74)	<0.001
HIV prevalence	0.00 (−0.20, 0.19)	0.99	−0.05 (−0.24, 0.15)	0.63
Year of vaccine introduction	−0.57 (−0.73, −0.41)	<0.001	−0.46 (−0.63, −0.29)	<0.001
Per capita GDP (logged)	−0.03 (−0.22, 0.17)	0.77	0.10 (−0.09, 0.30)	0.31
Savings rate (*s*)	0.02 (−0.18, 0.21)	0.84	0.25 (0.06, 0.44)	0.01
Growth rate of total factor productivity (*g*)	0.13 (−0.06, 0.33)	0.18	0.15 (−0.04, 0.35)	0.11
Growth rate of educational capital (*h*)	0.11 (−0.08, 0.31)	0.26	0.05 (−0.15, 0.24)	0.64
Output elasticity of physical capital (*α*)	−0.27 (−0.46, −0.08)	0.01	−0.29 (−0.48, −0.10)	0.01
Depreciation rate (*δ*)	0.03 (−0.17, 0.22)	0.79	−0.11 (−0.31, 0.08)	0.25

Note: For each country, the outcome represents the mean percentage increase in GDP for a given vaccination scenario relative to the “no-new-vaccine” counterfactual, cumulated over the 2028–2080 period. PRCCs quantify the direction and strength of the monotonic relationship between this outcome and each country characteristic, controlling for the effect of other characteristics.

GDP, gross domestic product; PRCC; partial rank correlation coefficient; TB, tuberculosis.

### Sensitivity analyses

Compared to the base–case assumption of 10-year protection, an adolescent/adult vaccine with lifelong duration of protection led to total gains to GDP of $2,518 ($1,140, 4,789) billion, a 56% increase compared to the base–case (Table 4). An adolescent/adult vaccine with 75% efficacy also produce larger GDP gains from vaccine introduction, estimated as $2,355 ($1,069, 4,387) billion (46% greater than the base–case). Compared to the base–case, the GDP gains produced by vaccine introduction were 26% lower in the low-coverage scenario and 18% higher in the high-coverage scenario. Compared to the base–case vaccination introduction and delivery scenario, the accelerated scale-up scenario produced greater GDP gains, $2,765 ($1,241, 5,229) billion, a 71% increase. Conversely, the “routine delivery-only” scenario produced much smaller GDP gains ($352 ($139, 710) billion), 78% lower than under the base–case.

**Table 4:** Gains to GDP due to adolescent/adult TB vaccines across 2028–2080 for 105 analyzed LMICs by vaccine characteristic and delivery scenario.

Scenario	Absolute gains in GDP (billions 2020 US dollars)	Percentage gain in GDP (%)
Base–case	1,618 (764, 2,988)	0.0326% (0.0266%, 0.0388%)
Lifelong duration of protection	2,518 (1,140, 4,789)	0.0484% (0.0403%, 0.0572%)
75% vaccine efficacy	2,355 (1,069, 4,387)	0.0452% (0.0377%, 0.0530%)
Low coverage	1,205 (527, 2,289)	0.0231% (0.0188%, 0.0276%)
High coverage	1,912 (843, 3,606)	0.0366% (0.0301%, 0.0436%)
Accelerated scale-up	2,765 (1,241, 5,229)	0.0532% (0.0445%, 0.0621%)
Routine delivery-only	352 (139, 710)	0.0067% (0.0049%, 0.0086%)
Rapid TB decline	55 (−77.6, 272)	0.0008% (−0.0022%, 0.0039%)

Note: Values in parentheses represent equal-tailed 95% uncertainty intervals.

GDP, gross domestic product; LMIC, low- and middle-income country; TB, tuberculosis.

With the alternative no-new-vaccine baseline representing faster incidence reductions through strengthening of current TB interventions (sufficient to meet 2035 End TB Strategy targets in each country), GDP gains from vaccine introduction were projected to be 97% smaller. For all relevant scenarios, the same relationships hold for the infant vaccine (Exhibit H in S1 Appendix).

Compared to the main analysis (estimated GDP gains of $1,687 ($816, 3,076) billion for the adolescent/adult vaccine, and $219 ($91, 422) billion for the infant vaccine) the first alternative specification of health services costs excluding patient-incurred costs resulted in estimated GDP gains that were 8% to 10% smaller (Exhibits I and J in S1 Appendix for adolescent/adult and infant vaccines, respectively) than estimated for the base–case specification. Under the second alternative specification of health services costs, excluding costs incurred by domestic governments resulted in estimated GDP gains that were 5% to 17% greater than estimated for the base–case specification (Exhibits K and L in S1 Appendix). Under the third alternative specification of health services costs, including costs attributed to international donors resulted in estimated GDP gains that were 10% to 22% lower than estimated for the base–case specification (Exhibits M and N in S1 Appendix).

Low-growth and high-growth specifications of GDP had a greater impact on the estimated economic impact of vaccine introduction compared to alternative specifications of health services costs. For the low-growth scenario, estimated GDP gains were 37% to 43% lower than estimated for the base–case specification (Exhibits O and P in S1 Appendix). Under the high-growth scenario, estimated GDP gains were 36% to 40% higher than estimated for the base–case specification (Exhibits Q and R in S1 Appendix).

Under the final alternative specification, with the economic impact of nonfatal health losses values represented as income losses rather than reductions in the labor supply, estimated GDP gains were 15% to 21% lower than estimated for the base–case specification (Exhibits S and T in S1 Appendix).

## Discussion

In this study, we estimated changes in the economic performance of 105 LMICs that would result from the introduction of novel TB vaccines, based on characteristics specified in the WHO PPCs [20], and promising evidence from ongoing clinical trials [40,41]. At a global level, both vaccine scenarios—accounting for the costs of introducing and implementing the new vaccination program—were shown to produce greater cumulative GDP in the modeled countries over the analytic period, with US$1,618 billion in economic gains estimated for the adolescent/adult vaccine and $207 billion for the infant vaccine. For both vaccine products, economic benefits were concentrated in countries with a high burden of TB and lagged relative to the timing of vaccine introduction, particularly for the infant vaccine. The magnitude of GDP gains was strongly associated with higher country TB incidence level and with earlier vaccine introduction. These factors contributed to the greater macroeconomic impacts seen among lower-middle-income countries (as compared to low-income and upper-middle-income country groups) and countries in the African and Southeast Asian regions. Despite vaccine introductions implemented between 2028 and 2047, the greatest impacts were projected closer to 2080 than 2050 for both vaccine products (Exhibits F and G in S1 Appendix) after vaccinees age into and contribute to the labor force.

As the costs of vaccination program were considered in the analysis (i.e., spending that could have otherwise been devoted to education or other investment), it was not automatically true that vaccine introduction would produce GDP gains. Indeed, in low TB incidence settings, the GDP gains were small or, in some cases, negative. For these countries, the health benefits of vaccination [6,9] might still justify the type of population-wide vaccination strategies considered in this analysis, or alternatively targeted vaccination strategies could be adopted, focusing on high-risk groups.

Study results were robust to most alternative analytic specifications for costs and economic growth. The sensitivity analyses in this group with the greatest impact involved changes in economic growth projections, with high- and low-growth projections leading to estimates of vaccine impact that were substantially larger and smaller, respectively, than estimated in the main analysis, illustrating the sensitivity of results to future economic conditions. Another sensitivity analysis that led to larger changes in the results was where we modified the approach taken to representing the macroeconomic impact of nonfatal illness. In this comparison, modeling nonfatal illness as directly reducing the labor supply (main analysis) produced economic impacts that were one-fifth greater than when we modeled nonfatal illness as producing additional costs for TB-affected households. Despite these differences, all the alternative analytic specifications suggested the economic gains of vaccine introduction would be substantial. On the other hand, assuming accelerated scale-up (i.e., vaccine introduction in 2025 at the specified coverage target for all countries) has much bigger impacts on economic gains compared to the base–case country-specific vaccine introduction timeline, which was based on patterns seen for the licensure-to-introduction timeline for previous vaccines such as pentavalent and pneumococcal conjugate vaccine [9,42].

It is important to note that the economic impacts estimated in this study would not be realized as an explicitly monetary gain, but instead as small, widely spread improvements in living standards within TB-affected countries. While small relative to overall changes in GDP, these economic gains are large relative to the investments needed to successfully develop TB vaccines. For example, the 2023–2030 Stop TB Global Plan includes $10 billion in research funding to successfully develop TB vaccines, less than 1% of the absolute GDP gains estimated for the adolescent/adult vaccine scenario [38]. Of course, the economic benefits of TB vaccine introduction would be substantially lagged relative to the timing of investments needed to develop these vaccines, which is a feature of vaccine interventions more generally. For this reason, decisions to invest in TB vaccines based on macroeconomic impacts must consider the longer time frame over which benefits will accrue, as well as the fact that these benefits will be largely invisible, despite their magnitude.

Historically, there have been limited macroeconomic analyses concerning tuberculosis for comparison to our analysis [43]. However, the cost–benefit ratio of novel TB vaccine introduction (calculated by dividing total the global economic gains of vaccine introduction by the cost of vaccine introduction and delivery) was relatively high compared to other public health interventions in studies that have examined economic benefits and costs including non-macroeconomic models [44], 14.2 for the adolescent/adult vaccine and 8.2 for the infant vaccine. Despite the trade-off between upfront investment in a new vaccine for future incremental gains in GDP, novel TB vaccination could provide a competitive return on investment.

A strength of this analysis is the approach used to estimated economic outcomes. Most studies estimating the economic impact of disease adopt a “Cost of Illness” approach, in which incidence of a given condition is multiplied by the range of per-episode costs borne by patients, governments, and broader society, in order to sum the total costs resulting from the disease. However, this “Cost of Illness” approach does not take into account the dynamic relationships between labor supply, capital accumulation, and economic growth. The macroeconomic model used for this analysis allows for these relationships. Moreover, for this analysis, we also extended this model to incorporate the effects of morbidity on individual productivity, a limitation noted in earlier applications [32].

This analysis has several limitations. Firstly, the macroeconomic model relies on projections of GDP that are inherently uncertain. Unpredictable natural disasters or conflicts could alter these projections and affect the conclusions drawn by this analysis. However, we attempted to address this limitation by examining both high- and low-growth scenarios for GDP and by allowing for stochastic changes in macroeconomic variables based on past performance. Secondly, we assumed a working age population of ages 15 to 69 contributing to the labor market. This assumption excludes older workers as well as informal caregivers that may affect labor force participation. Thirdly, we based the characteristics of new TB vaccines in our analyzed scenarios on the WHO PPCs [20], but a final product may differ in terms of effectiveness or duration of protection [1]. We also did not include any scenarios including booster doses following waning protection. Investigating such scenarios is a priority for future research. Future WHO recommendations regarding novel TB vaccination will follow the WHO-INTEGRATE framework [45–47], which stresses the relevance of a broad range of outcomes for decision-making. Ultimately, country-level decisions will determine the pace and scale of novel TB vaccine implementation, with these decisions being made according to the criteria that are important in the specific country context. Finally, we assumed that TB trends in the no-new-vaccine baseline would follow their historical trajectory. If there were aggressive scale-up of nonvaccine interventions (as envisaged by recent global TB strategy), this would reduce the incremental impact of a new vaccine [1].

To guide global efforts to accelerate TB control, it is critical to understand the full range of consequences of possible intervention options. If shown to be effective, TB vaccines promise to protect individuals from the risks associated with TB infection, leading to population-level reductions in TB burden [6], and TB-related catastrophic costs [11]. This study demonstrates that, under a range of assumptions, novel TB vaccines could have a further beneficial impact for TB-affected LMICs through increasing rates of economic growth. These impacts should be viewed not as alternative way of quantifying the benefits of vaccination, but as an additional benefit, which can be added to the value of the health gains and financial risk protections produced through reduced TB morbidity and mortality. This full range of outcomes should be considered when weighing the value of further investment in TB vaccine development.

## Supporting information

S1 Appendix Supporting information for “The potential impact of novel tuberculosis vaccine introduction on economic growth in low- and middle-income countries.” (DOCX)

## Abbreviations

AFR: African region
AMR: Region of the Americas
DALY: disability-adjusted life year
EMR: Eastern Mediterranean region
EPIC: Economic Projections of Illness and Cost
EUR: European region
GDP: gross domestic product
HPV: human papillomavirus
LMIC: low- and middle-income country
*Mtb*: *Mycobacterium tuberculosis*
PPCs: Preferred Product Characteristics
PRCC: partial rank correlation coefficient
SEAR: Southeast Asian region
TB: tuberculosis
WHO: World Health Organization
WPR: Western Pacific region
YLD: year lived with disability
