# Supplementary material for: The potential impact of novel tuberculosis vaccine introduction on economic growth in low- and middle-income countries: A modeling study
Source: PLoS Med. 2023 Jul 11;20(7):e1004252. doi: 10.1371/journal.pmed.1004252 (PMC10335702; doi:10.1371/journal.pmed.1004252)
Supplement: S1 Appendix — (DOCX) [file pmed.1004252.s001.docx]

**Supplementary Material for “The potential impact of novel tuberculosis vaccine introduction on economic growth in low- and middle-income countries”.**

**Table of Contents**

[Exhibit A. Epidemiological methods. 3](#_Toc136526961)

[Exhibit B. The CHEERS 2022 checklist. 23](#_Toc136526962)

[Exhibit C. International donor share of TB and HIV/AIDS spending based on development assistance for health spending estimates. 26](#_Toc136526963)

[Exhibit D. Unit cost inputs and assumptions (2020 USD). 30](#_Toc136526964)

[Exhibit E. Technical specifications of the macroeconomic model 31](#_Toc136526965)

[Exhibit F. Cumulative absolute gains to gross domestic product (GDP, billions US$2020) due to adolescent/adult tuberculosis vaccines by decade. 35](#_Toc136526966)

[Exhibit G. Cumulative absolute gains to gross domestic product (GDP, billions US$2020) due to infant tuberculosis vaccines by decade. 36](#_Toc136526967)

[Exhibit H. Gains to gross domestic product (GDP) due to infant tuberculosis (TB) vaccines across 2028–2080 for 105 analyzed low- and middle-income countries by vaccine characteristic and delivery scenario. 37](#_Toc136526968)

[Exhibit I. Gains to gross domestic product (GDP) due to adolescent/adult tuberculosis vaccines across 2028–2080: health services costs included government-level and excluded patient- and international-donor-level. 38](#_Toc136526969)

[Exhibit J. Gains to gross domestic product (GDP) due to infant tuberculosis vaccines across 2028–2080: health services costs included government-level and excluded patient- and international-donor-level. 39](#_Toc136526970)

[Exhibit K. Gains to gross domestic product (GDP) due to adolescent/adult tuberculosis vaccines across 2028–2080: health services costs included patient-level and excluded government- and international-donor-level. 40](#_Toc136526971)

[Exhibit L. Gains to gross domestic product (GDP) due to infant tuberculosis vaccines across 2028–2080: health services costs included patient-level and excluded government- and international-donor-level. 41](#_Toc136526972)

[Exhibit M. Gains to gross domestic product (GDP) due to adolescent/adult tuberculosis vaccines across 2028–2080: health services costs included all levels (government, patient, international donor). 42](#_Toc136526973)

[Exhibit N. Gains to gross domestic product (GDP) due to infant tuberculosis vaccines across 2028–2080: health services costs included all levels (government, patient, international donor). 43](#_Toc136526974)

[Exhibit O. Gains to gross domestic product (GDP) due to adolescent/adult tuberculosis vaccines across 2028–2080: low-growth scenario. 44](#_Toc136526975)

[Exhibit P. Gains to gross domestic product (GDP) due to infant tuberculosis vaccines across 2028–2080: low-growth scenario. 45](#_Toc136526976)

[Exhibit Q. Gains to gross domestic product (GDP) due to adolescent/adult tuberculosis vaccines across 2028–2080: high-growth scenario. 46](#_Toc136526977)

[Exhibit R. Gains to gross domestic product (GDP) due to infant tuberculosis vaccines across 2028–2080: high-growth scenario. 47](#_Toc136526978)

[Exhibit S. Gains to gross domestic product (GDP) due to adolescent/adult tuberculosis vaccines across 2028–2080: scenario with alternative approach to modelling the consequences of TB morbidity outcomes. 48](#_Toc136526979)

[Exhibit T. Gains to gross domestic product (GDP) due to infant tuberculosis vaccines across 2028–2080: scenario with alternative approach to modelling the consequences of TB morbidity outcomes. 49](#_Toc136526980)

[References 50](#_Toc136526981)

# Exhibit A. Epidemiological methods.

The subsequent pages provide relevant details and methods regarding the underlying epidemiological model and vaccine delivery scenarios from Clark et al. [1].

**A.1. Tuberculosis natural history structure**

The core natural history model is specified in Figure A.1. Those with no previous exposure or infection with *Mtb* [Uninfected-Naive (U_N_)] could become infected at rate
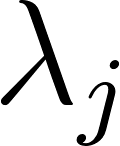
 and progress to an Infection-Fast (I_F_) class following initial infection. From Infection-Fast, three possible pathways were possible: (i) Fast progression to Subclinical Disease (D_S_), where individuals are infectious with a reduced infectiousness compared to clinical tuberculosis, but display no symptoms of tuberculosis disease [2]; (ii) self-clearance to Uninfected-Cleared (U_C_), where individuals are no longer infected with *Mtb* and therefore are not at risk of progression to tuberculosis disease without reinfection [3]; or (iii) continue to remain latently infected with a risk of reactivation and progression to disease, albeit at a lower rate than Infection-Fast, by transitioning to the Infection-Slow (I_S_) class. Those in the Infection-Slow class could self-clear to the Uninfected-Cleared class, be reinfected and return to the Infection-Fast class or reactivate their infection and progress to Subclinical Disease.

Once in the Subclinical Disease class, individuals could naturally cure (without treatment) to the Resolved (R) class, or progress to Clinical Disease (D_C_), where individuals are infectious and display symptoms of tuberculosis disease. Treatment initiation from Clinical Disease to On-Treatment (T) began in 1960 and increased following a sigmoid curve to 2019, with average treatment duration assumed to be six months [4,5]. Treatment completions transitioned to the Resolved class and treatment non-completions returned to Clinical Disease. Deaths occurring on-treatment and in clinical disease counted toward the total number of tuberculosis deaths during the year. Those with clinical disease could also naturally cure to the resolved class. Individuals in the Resolved class could be reinfected or relapse to Subclinical. We assumed that the infection and resolved classes are partially protected against reinfection [6,7]. In those who have self-cleared, we assumed the level of protection against reinfection is half of the protection against reinfection for the infection and resolved classes. Age was modelled in single years from ages 0 to 79 and aggregated into two categories for ages 80 to 89, and ages 90 to 99. Births and ageing occurred at the beginning of each year.

**A.2. HIV and ART structure description**

To account for the influences of human immunodeficiency virus (HIV) and antiretroviral therapy (ART) on the risk of infection with *Mtb* and progression to tuberculosis disease [6,8], we implemented an HIV structure (shown in Figure A.2) composed of 3 compartments: HIV uninfected [HIV0], people living with HIV (PLHIV) not on ART [HIV1], and PLHIV on ART [ART]. HIV uninfected individuals were diagnosed with HIV and moved from the HIV0 compartment to the HIV1 compartment with rate [
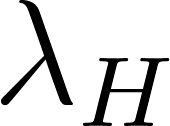
](https://www.codecogs.com/eqnedit.php?latex=%5Clambda_H#0). Within the HIV1 compartment, there is a higher risk of tuberculosis progression and an increased tuberculosis mortality rate compared to the HIV0 compartment. PLHIV are initiated on treatment with ART from HIV1 following a sigmoid trend. The increases in tuberculosis mortality rate and tuberculosis progression are reduced while on ART compared to HIV1, but still higher than in HIV0. ART also reduces the HIV mortality rate.

The separate stratum was included to dynamically model the tuberculosis-HIV co-epidemic if the proportion of tuberculosis cases among people living with HIV (PLHIV) was greater than or equal to 15%, and if the HIV prevalence in the country was greater than 1% [9,10]. Countries incorporating the additional HIV structure are listed in Table A.1.


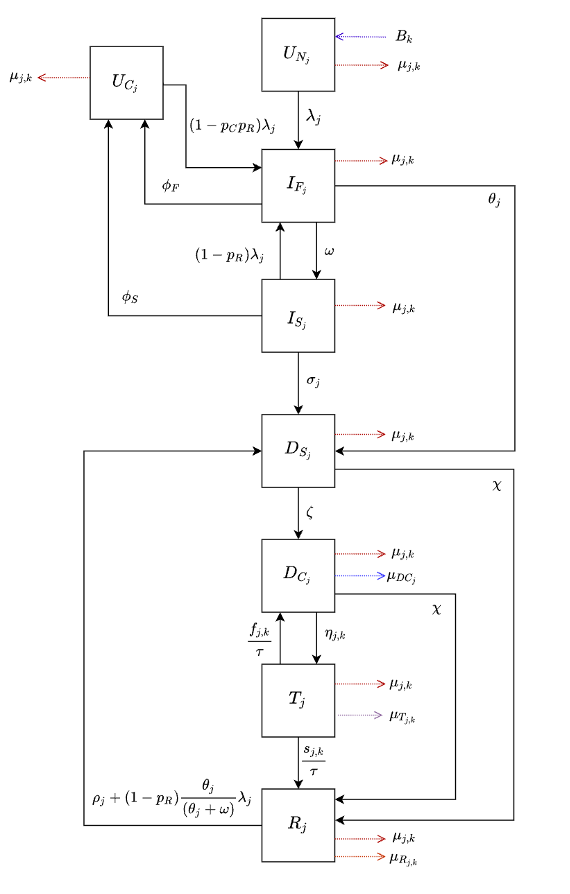


**Figure A.1. Tuberculosis natural history model**

*Subscript j represents parameters that vary by age, and subscript k represents parameters that vary over time.*

*Abbreviations: D_C_ = Clinical Disease; D_S_ = Subclinical Disease; I_F_ = Infection-Fast; I_S_ = Infection-Slow; R = Resolved; T = On-Treatment; U_C_ = Uninfected-Cleared; U_N_ = Uninfected-Naïve.*


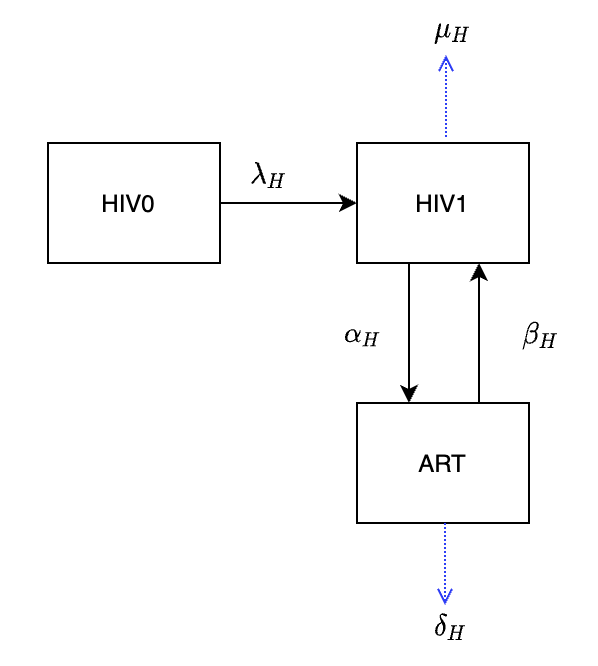


**Figure A.2. HIV and ART structure.**

*Abbreviations: ART = People living with HIV on ART; HIV0 = HIV uninfected; HIV1= People living with HIV not on ART.*

**Table A.1.** **Countries incorporating the HIV structure with their corresponding HIV prevalence and proportion of tuberculosis cases among PLHIV.**

| **Country** | **HIV Prevalence (%)** | **Proportion of tuberculosis cases among PLHIV (%)** |
| --- | --- | --- |
| Botswana | 16.5 | 48.6 |
| Central African Republic | 2.1 | 25.4 |
| Côte d’Ivoire | 1.7 | 17.5 |
| Cameroon | 2.0 | 26.8 |
| Gabon | 2.3 | 32.8 |
| Ghana | 1.1 | 20.8 |
| The Gambia | 1.2 | 17.7 |
| Guinea-Bissau | 2.1 | 31.3 |
| Equatorial Guinea | 4.8 | 26.5 |
| Guyana | 1.1 | 19.0 |
| Kenya | 2.9 | 26.2 |
| Lesotho | 16.0 | 61.6 |
| Mozambique | 7.2 | 33.8 |
| Malawi | 5.9 | 46.6 |
| Namibia | 8.4 | 32.5 |
| Rwanda | 1.8 | 21.1 |
| Eswatini | 17.4 | 60.1 |
| Togo | 1.5 | 16.2 |
| Tanzania | 2.9 | 23.6 |
| Uganda | 3.4 | 39.0 |
| South Africa | 12.8 | 58.0 |
| Zambia | 6.7 | 46.2 |
| Zimbabwe | 9.6 | 59.8 |

**A.3. Calibration methodology**

The model was fitted to epidemiologic calibration targets using history matching with emulation, implemented using the hmer R package [11,12]. If countries were unable to find at least 1000 fully fitted parameter sets using this method, they were subsequently assessed using an Approximate Bayesian Computation using Markov Chain Monte Carlo method (ABC-MCMC). ABC-MCMC was conducted using the easyABC package in R, modified by Sebastian Funk, Gwenan Knight, and the Tuberculosis Modelling group at LSHTM for adaptive sampling and to accept seeded parameter values [11,13]. We used parameter sets with the maximum number of targets fitted using history matching with emulation as a starting seed, with the ABC-MCMC algorithm continuously adapting using the last 1000 points and the noise factor set to 0.0001.

Analysis was performed on 105 countries from the 135 total low- and middle-income countries identified based on 2019 World Bank Income groups. There were 20 countries excluded from the initial calibration attempt due to missing crucial data required to attempt calibration, and 10 countries which were unable to be calibrated (could not find a parameter set that matched all targets using both history matching with emulation as well as ABC-MCMC). Reasons for exclusion from the final list of calibrated countries are provided in Table A.2.

**Table A.2. Reasons for exclusion from the final list of calibrated countries.**

| **Country** | **Reason for Exclusion** |
| --- | --- |
| Algeria | Did not calibrate |
| American Samoa | Missing multiple critical epidemiological data for calibration, no contact matrices available |
| Belize | No case notification or incidence data for children |
| Bosnia and Herzegovina | Did not calibrate |
| Cabo Verde | Did not calibrate |
| Comoros | No case notification data |
| Democratic Republic of the Congo | No case notification data by age |
| Republic of the Congo | No population estimates |
| Democratic People's Republic of Korea | Missing multiple critical epidemiological data for calibration |
| Djibouti | No case notification data by age |
| Dominica | Missing multiple critical epidemiological data for calibration, no contact matrices available |
| Guinea-Bissau | Did not calibrate |
| Guyana | Did not calibrate |
| Federated States of Micronesia | Missing multiple critical epidemiological data for calibration, no contact matrices available |
| Grenada | Missing multiple critical epidemiological data for calibration, no contact matrices available |
| Haiti | Missing 2020 contact matrix |
| Jamaica | Did not calibrate |
| Kiribati | Missing 2020 contact matrix |
| Kosovo | Missing multiple critical epidemiological data for calibration |
| Lebanon | Missing 2020 contact matrix |
| Marshall Islands | Missing multiple critical epidemiological data for calibration, no contact matrices available |
| North Macedonia | Did not calibrate |
| Samoa | No case notification or incidence data for children |
| Somalia | No contact matrices available |
| St. Lucia | No case notification or incidence data for children |
| St. Vincent and the Grenadines | Did not calibrate |
| Tonga | Did not calibrate |
| Turkmenistan | Did not calibrate |
| Tuvalu | No contact matrices available |
| West Bank and Gaza | Missing multiple critical epidemiological data for calibration |

**A.4. Vaccine profile**

The vaccine profile for an adult/adolescent vaccine and infant vaccine were based on the WHO Preferred Product Characteristics for New Tuberculosis vaccines [14], and are outlined in Table A.3 below.

**Table A.3.** **WHO Preferred Product Characteristics for New Tuberculosis Vaccines.**

| **Vaccine** | **Host infection status at time of vaccination required for efficacy** | **Effect type** | **Vaccine efficacy** | **Duration of protection** |
| --- | --- | --- | --- | --- |
| Adolescent / Adult | Pre- and post-infection | Prevention of disease | 50% | Lifelong |
|  |  |  |  | 10 years |
| Infant | Pre-infection | Prevention of disease | 80% | Lifelong |
|  |  |  |  | 10 years |

Vaccine efficacy was assumed to be the same in both PLHIV and HIV-naïve recipients in countries incorporating the HIV structure, and in both younger age groups and older adults. The vaccine was assumed to have the same impact on preventing drug-susceptible and drug-resistant tuberculosis as specified in the WHO PPCs [14]. As we were modelling a prevention of disease vaccine, there was no direct impact on *Mtb* transmission or the force of infection.

We assumed duration of protection was 10 years on average, in addition to a sensitivity analysis with lifelong duration of protection. The shape of waning immunity was modelled as an exponential distribution, based on similar shapes for waning vaccine immunity of BCG [15] and other vaccines [16,17].

**A.5. Vaccine delivery scenarios**

The infant vaccine was implemented in two scenarios, and, separately, the adolescent/adult vaccine was implemented in three scenarios. The *Basecase* and *Accelerated Scale-up* scenarios included routine single-dose neonatal vaccination for the infant vaccine (85% coverage), and routine single-dose vaccination of 9-year-olds (80% coverage) with a one-time vaccination campaign for ages ten and older (70% coverage) for the adolescent/adult vaccine. The *Routine Only* scenario (adolescent/adult vaccine only) was introduced through routine 9-year-old vaccination only (i.e., no campaign). Specifics of the infant and adolescent/adult vaccine scenarios are provided in Table A.4.

**Table A.4. Vaccine scenarios for the infant and adolescent/adult vaccines**.

| **Characteristics** | **Infant Vaccine Scenarios** | | **Adolescent/Adult Vaccine Scenarios** | | |
| --- | --- | --- | --- | --- | --- |
|  | ***Basecase*** | ***Accelerated***  ***Scale-up*** | ***Basecase*** | ***Accelerated***  ***Scale-up*** | ***Routine Only*** |
| **Ages Targeted** | *Neonatal:*  Routine | *Neonatal:*  Routine | *Age 9:* Routine  *Ages 10+:* One-time vaccination campaign over 5 years | *Age 9:* Routine    *Ages 10+:* One-time vaccination campaign in 2025 | *Age 9:* Routine |
| **Introduction Year** | Country-specific | 2025 | Country-specific | 2025 | Country-specific |
| **Vaccine Rollout  Trend** | 5-year linear scale-up to coverage | Instant scale-up to coverage | 5-year linear scale-up to coverage | Instant scale-up  to coverage | 5-year linear scale-up to coverage |
| **Target Coverage *(Low/Med/High)*** | 75% / 85% / 95% | | Age 9: 70% / 80% / 90%  Ages 10+: 50% / 70% / 90% | | |

**A.6. Country-specific introduction years**

In the *Basecase* and *Routine Only* scenarios, vaccines were introduced in country-specific introduction years between 2028 and 2047. The year 2028 was selected as the earliest country-specific introduction year to align with the anticipated completion and availability of results from TB vaccine candidate trials based on expert consultation and analysis. Country-specific introduction years were calculated for all 135 LMICs based on the 2019 World Bank Income groups. To calculate the specific year of introduction, countries were divided into two general categories: those procuring with support from Gavi, the Vaccine Alliance, and those self-procuring. Determination of country status was based on eligibility information posted on Gavi’s website [18]. Countries transitioning from Gavi support are able to benefit from Gavi pricing and incremental financing for a period of 5–10 years. For countries that have already initiated the period of transition by 2019, this window will have largely ended by the time of tuberculosis vaccine availability through Gavi. As such, these countries were categorized as self-procuring countries. Countries that have not yet commenced transition, including India and Nigeria, were categorised as Gavi supported countries, given the long grace period post-commencement of transition. For more information, please see Gavi, <https://www.gavi.org/types-support/sustainability/transition> (retrieved December 1, 2020).

Through a consultative process with experts from WHO, Gavi, PATH, PDVAC, CHAI, and industry partners, factors influencing likelihood of being an early or late adopter were identified for both Gavi and self-procuring countries. Identified factors include disease burden, immunization capacity, and early adopter status. Country-specific registration timelines and commercial prioritization were also deemed important determinants of introduction timing for self-procuring countries.

*Additional factors for Gavi countries:* For countries procuring through Gavi, timelines for introduction are also influenced by Gavi processes. Prior to offering a new vaccine, Gavi requires that products be licensed, included in Gavi’s Vaccine Investment Strategy, reviewed by SAGE, recommended in a WHO position paper, WHO prequalified, and approved for procurement by Gavi (Table A.5). In addition, time for country application processing, contracting, and delivery must be factored. Through consultations, it was determined that a baseline time of roughly two years post licensure would be needed for Gavi processes prior to first country introduction, assuming several steps advance in parallel.

**Table A.5. Timelines for Gavi processes post licensure.**

|  | **Cumulative additional time (years)** | | |
| --- | --- | --- | --- |
| **Activities post licensure** | **Low End** | **High End** | **Average** |
| WHO PQ | 0.25 | 1.00 | 0.63 |
| SAGE Policy Review & WHO Position Paper | 0.25 | 0.50 | 0.38 |
| Gavi Decision | 0.25 | 0.50 | 0.38 |
| National review & Country applications | 0.25 | 0.75 | 0.50 |
| Contracting & delivery | 0.25 | 0.50 | 0.38 |
| **Years** | **1.25** | **3.25** | **2.25** |

*Weight of criteria, indicators, and scoring*: Differential weight was assigned to criteria based on their relative impact on the order of country adoption. This weight varied for self-procuring and Gavi countries (Table A.6).

**Table A.6. Weight of criteria influencing order of country adoption.**

| **Criteria** | **Self-procuring countries** | **Gavi countries** |
| --- | --- | --- |
| Disease burden | 30% | 45% |
| Immunization capacity | 15% | 30% |
| Early adopter/leader | 15% | 25% |
| Lack of regulatory barriers | 15% | NA |
| Commercial prioritization | 25% | NA |

The following indicators were used to measure each of the variables identified in Table A.7.

**Table A.7. Indicators of criteria influencing order of country adoption.**

| **Criteria** | **Indicator** |
| --- | --- |
| **Disease burden** | Tuberculosis incidence |
| **Immunization capacity** | Proportion receiving 3 doses of DPT3 among infants 1 years of age (The percent of infants receiving 3 doses DPT3 is commonly used as a proxy for assessing immunization infrastructure) |
| **Lack of regulatory barriers** | Signatories to WHO PQ or SRA collaborative registration scheme  Lack of requirements for additional local clinical trial data |
| **Early adopter/leader** | Time to policy adoption of universal Xpert MTB/RIF screening for presumed tuberculosis cases  Time to adoption of HPV |
| **Commercial prioritization** |  |
| *Ability to finance vaccines* | GDP per capita |
| *Political will to address tuberculosis* | Spending per tuberculosis case |
| *Market potential* | Population |

To standardize across these varied metrics, a point value ranging from 1–5 per criteria was assigned, with a score of 1 correlating with an earlier adopter and score of 5 correlating with a later adopter.

*Continuous variable*s such as disease burden or population were divided into quintiles. Those in the highest quintile were assigned a score of 1, those in the second highest quintile received a score of 2, and so forth. *Categorical variables* such as registration or early adopter status were scored based on whether countries met fixed criteria. For instance, countries that are signatories of WHO PQ or SRA collaborative registration schemes were assigned a score of 1. Those that are not signatories and have requirements for additional clinical trial data in local populations received a score of 5.

Scores were then weighted as reflected in Table A.6 and aggregated into a composite score to determine countries’ relative position in the queue of introductions.

Assumptions for the pace of introduction—i.e., how many countries per year would introduce the product and what the scale up curve might look like— was informed with data from pneumococcal vaccine (PCV) scale-up [19]. The percent of countries adopting each year (year 1 to year 12) for PCV was calculated. These annual percentages were then applied to tuberculosis vaccine scale up (based on a total n=135 countries: 78 self-procuring countries and 57 Gavi countries). The first year of tuberculosis vaccine scale up was estimated to be 2028, with Gavi countries following a similar scale up trajectory but delayed by two years due to required Gavi lead time for processing new vaccines (Table A.5). Because PCV data is only available for 12 years, data was extrapolated for years 13 to 20 of tuberculosis vaccine roll out at a steady state. Country introduction timelines were adjusted—where applicable—to group countries with the same composite score in the same year of adoption. The cumulative number of countries introducing the vaccine by year is shown in Figure A.3.

**
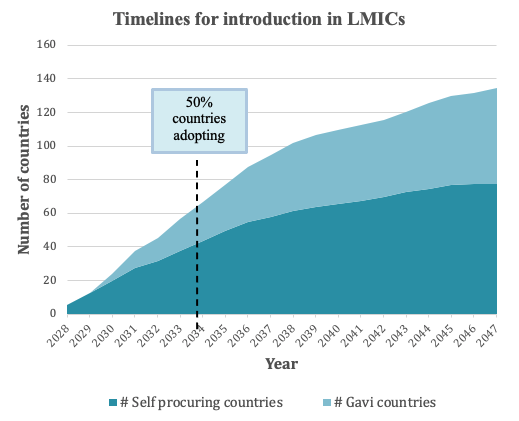
**

**Figure A.3.** **Assumed cumulative number of countries introducing a novel vaccine per year.**

**A.7. Vaccine coverage targets.**

For each vaccine implementation scenario, low, medium, and high coverage targets for 5 years post-introduction were evaluated. The medium coverage target for the routine infant vaccination was 85%, based on the 2019 DTP3 (diphtheria, tetanus toxoid, and pertussis) average coverage level according to the WHO and UNICEF estimates of national immunisation coverage, with 10% uncertainty (low coverage = 75%, high coverage = 95%) [19]. Routine adolescent vaccination assumed a medium coverage target of 80% aligning with HPV coverage in South Africa combined with aggregated secondary school enrolment in China and India as assumed in Harris 2020 [20], also with 10% uncertainty targets (low coverage = 70%, high coverage = 90%). The medium coverage target for the adolescent/adult campaign was 70% aligning with the lower bound of the MenAfriVac campaigns in sub-Saharan Africa as assumed in Harris 2020 [20], with a wider uncertainty of 20% (low coverage = 50%, high coverage = 90%).

In the *Accelerated Scale-up* implementation, the 5-year coverage targets were achieved instantly in year 1, while in the *Basecase* and *Routine Only* implementations, the scale-up to coverage occurred linearly over 5 years.

**A.8. Calibrated *No-New-Vaccine* baseline trends.**

Here we show the tuberculosis incidence rates plotted from 2000–2050 for the selected grouping for reporting model outcomes. In Figure A.4, looking by WHO region, we see the incidence rates are highest in AFR and SEAR, and lowest in AMR and EUR. In Figure A.5, we show the incidence rate trends by income group. Both incidence and mortality rates follow a trend with the highest estimated medians in lower-middle-income countries, followed by low-income countries and high-income countries, which aligns with the expectation of burden within each region. In Figure A.6, we compare incidence rates between countries included on the WHO high TB burden list and all other countries modelled, and as expected, higher values are predicted for countries on the high TB burden list.

**Figure A.4. Tuberculosis incidence rates for the *No-New-Vaccine* baseline by WHO region.**


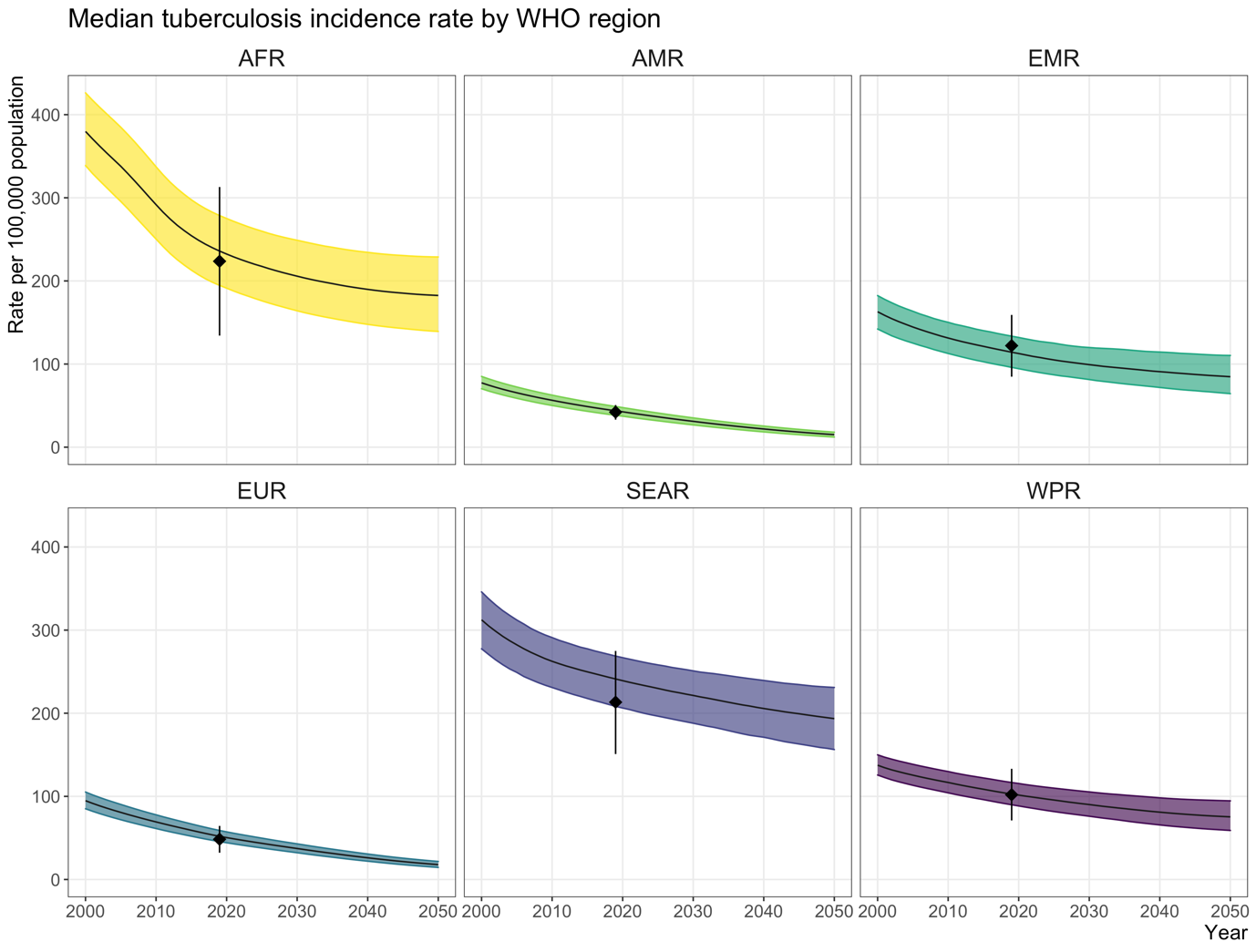


Note: The black diamond is the WHO median estimate of the incidence in 2019 for the 105 modelled LMICs by WHO region with 95% uncertainty range. The black line is the model estimated median incidence rate, with shaded 95% uncertainty ranges. AFR = WHO African Region, AMR = WHO Region of the Americas, EMR = WHO Eastern Mediterranean Region, EUR = WHO European Region, SEAR = WHO South-East Asian Region, WPR = WHO Western Pacific Region.

**Figure A.5. Tuberculosis incidence rates for the *No-New-Vaccine* baseline by income group.**


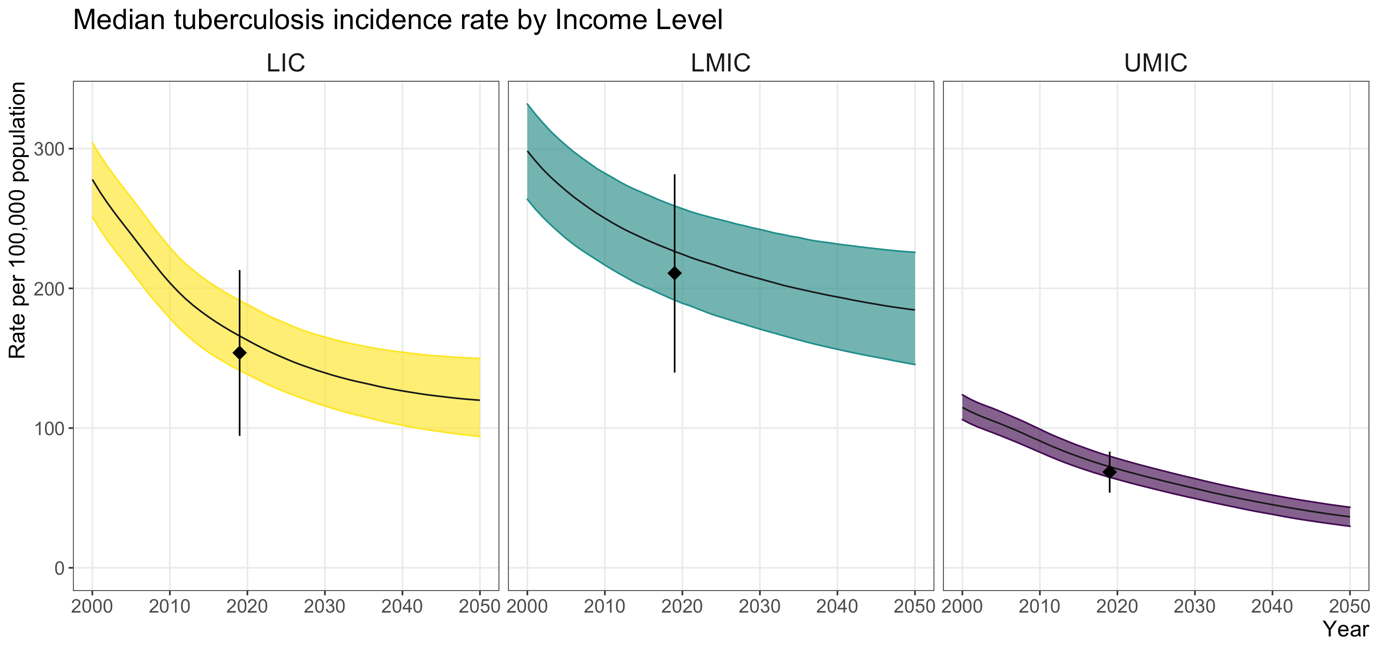


Note: The black diamond is the WHO median estimate of the incidence rate in 2019 for the 105 modelled LMICs by income group with 95% uncertainty range. The black line is the model estimated median incidence rate, with shaded 95% uncertainty ranges. LIC = low-income countries, LMIC = lower middle-income countries, UMIC = upper middle-income countries.

**Figure A.6. Tuberculosis incidence rates for the *No-New-Vaccine* baseline for the countries included on the WHO high-TB-burden list and for all other countries modelled.**


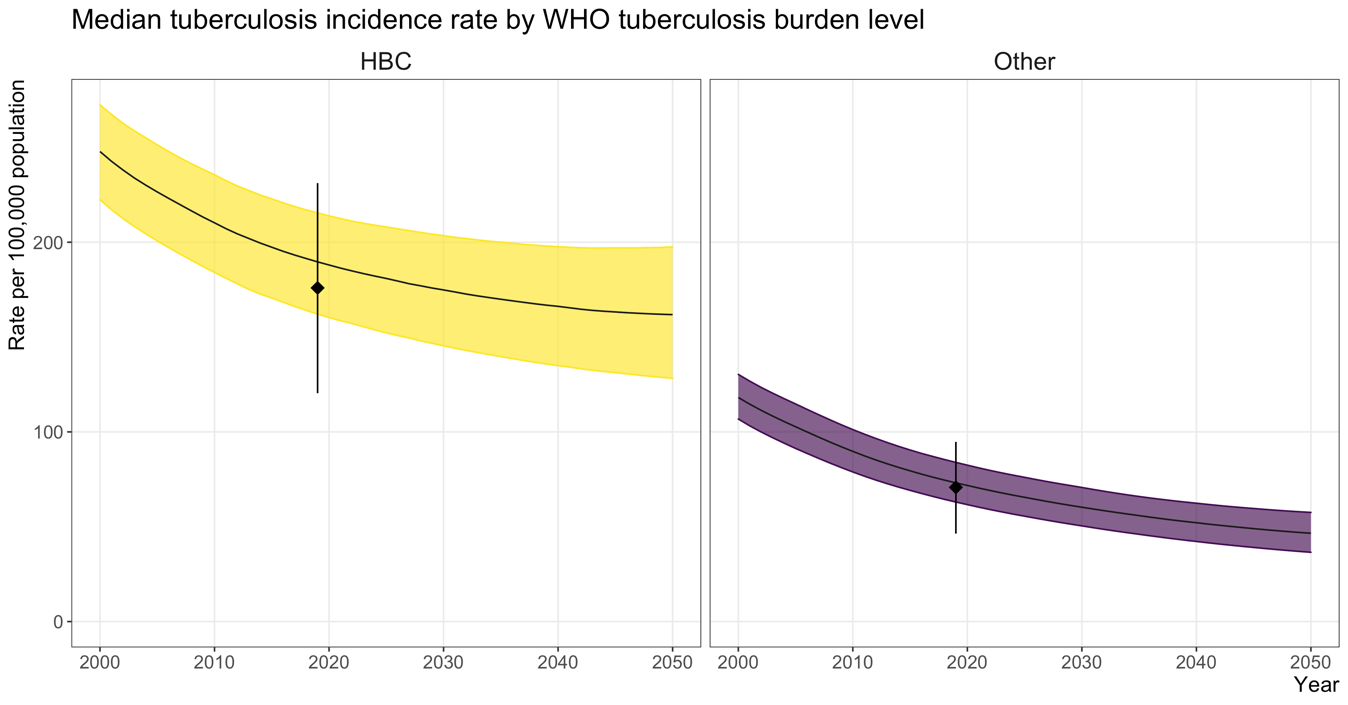


Note: The black diamond is the WHO median estimate of the incidence rate in 2019 for the 105 modelled LMICs by burden level with 95% uncertainty range. The black line is the model estimated median incidence rate, with shaded 95% uncertainty ranges. HBC = high burden countries.

**A.9. Model Parameters and Data Sources.**

Parameters used in the natural history model structure and the HIV and ART model structure are provided in Table A.8 below, along with their definitions, sources, and information on whether the parameter is fixed or varied (as well as whether they are varied by age or time) during calibration. The parameter ranges provided for the tuberculosis natural history parameters are priors fitted during calibration in a Bayesian analysis. We assume that all values within the prior range are equally likely. The prior ranges were pre-specified based on literature review and were reviewed as new data became available.

**Table A.8. Demographic and tuberculosis natural history parameters and definitions.**

| **Description** | **Units** | **Symbol** | **Prior** | **Fixed or Varying During Calibration** | **Age Varying** | **Time Varying** | **Source** |
| --- | --- | --- | --- | --- | --- | --- | --- |
| ***Births and deaths (excluding on-treatment mortality)*** | | | | | | | |
| Birth rate | Per year | [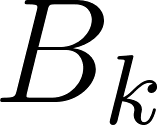](https://www.codecogs.com/eqnedit.php?latex=B_k#0) | United Nations World Population Prospects population estimates and projections | Fixed | No | Yes | [21] |
| Background mortality rate | Per year | [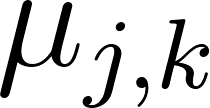](https://www.codecogs.com/eqnedit.php?latex=%5Cmu_%7Bj%2Ck%7D#0) | Calculated in the model from United Nations population estimates and projections | Fixed | Yes, age specific mortality rates from demographic dataset | Yes | [21] |
| Mortality rate for clinical tuberculosis disease | Per person  per year | [***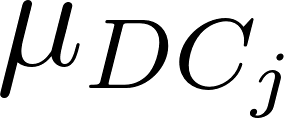***](https://www.codecogs.com/eqnedit.php?latex=%5Cmu_%7BDC_j%7D#0) | (0–0·178) | Varying | Yes, value for children is greater than value for adults | No | [22] |
| Mortality rate post-tuberculosis disease | Per person  per year | [*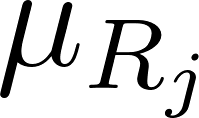*](https://www.codecogs.com/eqnedit.php?latex=%5Cmu_%7BR_j%7D#0) | $0\cdot22\mu_{j,k}$ | Fixed relationship | Yes, because [$\mu_{j,k}$](https://www.codecogs.com/eqnedit.php?latex=%5Cmu_%7Bj%2Ck%7D#0) varies | Yes, because [$\mu_{j,k}$](https://www.codecogs.com/eqnedit.php?latex=%5Cmu_%7Bj%2Ck%7D#0) varies | [23] |
| ***Natural history*** | | | | | | | |
| Force of infection | Per year | [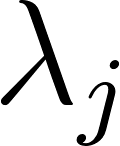](https://www.codecogs.com/eqnedit.php?latex=%5Clambda_j#0) | Fitted | Fixed equation | Yes, age specific contact rates^9^ | No | *Calculated* |
| Probability of transmission per infectious contact | - | [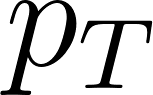](https://www.codecogs.com/eqnedit.php?latex=p_T#0) | (0–0·0068) | Varying | No | No | *Assumed* |
| Fraction of total tuberculosis disease that is extrapulmonary | - | [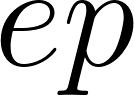](https://www.codecogs.com/eqnedit.php?latex=ep#0) | Country-specific average of previous 3 years | Fixed | No | No | [24,25] |
| Infectiousness of subclinical relative to clinical tuberculosis | - | [*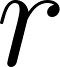*](https://www.codecogs.com/eqnedit.php?latex=r#0) | 0·80 | Fixed | No | No | [26] |
| Rate of self-clearance  from I_F_ to U_C_ | Per person  per year | [*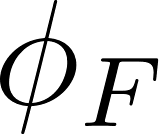*](https://www.codecogs.com/eqnedit.php?latex=%5Cphi_F#0) | 0·00000140 | Fixed | No | No | [3] |
| Rate of self-clearance  from I_S_ to U_C_ | Per person  per year | [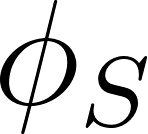](https://www.codecogs.com/eqnedit.php?latex=%5Cphi_S#0) | (0·0254–0·0467) | Varying | No | No | [3] |
| Rate of fast progression to disease, by age | Per person  per year | [*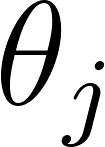*](https://www.codecogs.com/eqnedit.php?latex=%5Ctheta_j#0) | (0·0696–0·111) | Varying | Yes, value for children is less than value for adults | No | [3] |
| Rate from I_F_ to I_S_ | Per person  per year | [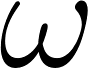](https://www.codecogs.com/eqnedit.php?latex=%5Comega#0) | 0·5 | Fixed | No | No | *Defined* |
| Rate of reactivation  from I_S_, by age | Per person  per year | [*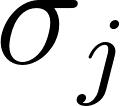*](https://www.codecogs.com/eqnedit.php?latex=%5Csigma_j#0) | (0·000135–0·00113) | Varying | Yes, value for children is less than value for adults | No | [3] |
| Rate of progression  from D_S_ to D_C_ | Per person  per year | [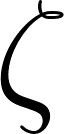](https://www.codecogs.com/eqnedit.php?latex=%20%5Czeta%20#0) | (0–1) | Varying | No | No | *Assumed* |
| Rate of natural cure  from D_C_ and D_S_ | Per person  per year | [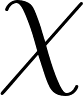](https://www.codecogs.com/eqnedit.php?latex=%5Cchi#0) | (0·1–0·25) | Varying | No | No | [27,28] |
| Rate of relapse from R,  by age | Per person  per year | [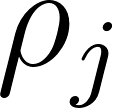](https://www.codecogs.com/eqnedit.php?latex=%5Crho_j#0) | (0·0001–0·07) | Varying | Yes, value for children is less than value for adults | No | [29-31] |
| ***Treatment outcome parameters*** | | | | | | | |
| Treatment duration | Number of years | [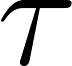](https://www.codecogs.com/eqnedit.php?latex=%20%5Ctau%20#0) | 0·5 | Fixed | No | No | [4,5] |
| Rate of on-treatment mortality | Per person  per year | [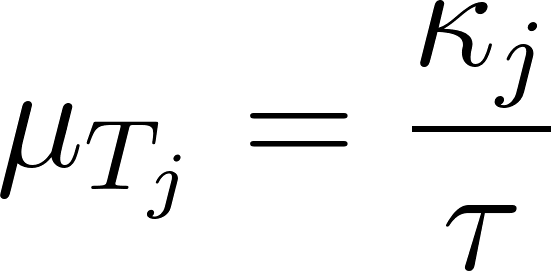](https://www.codecogs.com/eqnedit.php?latex=%5Cmu_%7BT_j%7D%20%3D%20%5Cfrac%7B%5Ckappa_j%7D%7B%5Ctau%7D#0) | Country-specific | Varying | Yes, value for children greater than value for adults | Yes | [32] |
| Rate of treatment completion | Per person  per year | [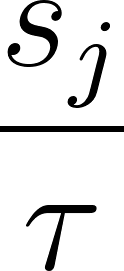](https://www.codecogs.com/eqnedit.php?latex=%5Cfrac%7Bs_j%7D%7B%5Ctau%7D#0) | Country-specific | Fixed equation | Yes, indirectly scaled by *s_Age_* | Yes | [3] |
| Rate of treatment  non-completion | Per person  per year | [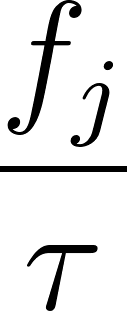](https://www.codecogs.com/eqnedit.php?latex=%5Cfrac%7Bf_j%7D%7B%5Ctau%7D%20#0) | Country-specific | Fixed equation | Yes, indirectly scaled by *s_Age_* | Yes | [3] |
| ***Protection parameters*** | | | | | | | |
| Protection from  reinfection for I_S_, I_F_, R | - | [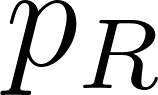](https://www.codecogs.com/eqnedit.php?latex=%20p_R%20#0) | (0·6–0·85) | Varying | No | No | [7,27,28,33,34] |
| Relative protection  from reinfection for self-clearance compared to [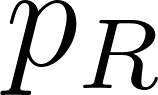](https://www.codecogs.com/eqnedit.php?latex=%20p_R%20#0) | - | [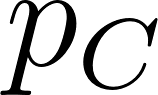](https://www.codecogs.com/eqnedit.php?latex=p_C#0) | 0·50 | Fixed | No | No | *Assumed* |
| SES parameter | - | [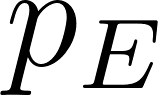](https://www.codecogs.com/eqnedit.php?latex=p_E#0) | (0–1) | Varying | No | No | *Assumed* |
| ***HIV parameters*** | | | | | | | |
| HIV incidence rate fitting factor | - | [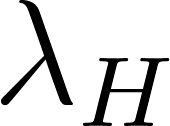](https://www.codecogs.com/eqnedit.php?latex=%5Clambda_H#0)fit | (0–300) | Varying | No | No | *Fitted* |
| Rate of ART initiation fitting factor | - | [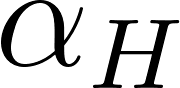](https://www.codecogs.com/eqnedit.php?latex=%5Calpha_H#0)fit | (0–7000) | Varying | No | No | *Fitted* |
| Rate of ART discontinuation | Per year | [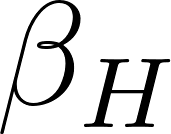](https://www.codecogs.com/eqnedit.php?latex=%5Cbeta_H#0) | 0·074 | Fixed | No | No | [35,36] |
| Mortality rate from  HIV not on ART | Per year | [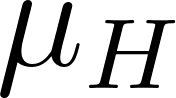](https://www.codecogs.com/eqnedit.php?latex=%5Cmu_H#0) | 0·10 | Fixed | No | No | [37] |
| Mortality rate from  HIV on ART | Per year | [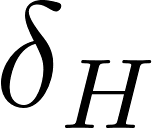](https://www.codecogs.com/eqnedit.php?latex=%5Cdelta_H#0) | 0·026 | Fixed | No | No | [38] |
| Relative increase in progression rate for HIV_1_ | - | [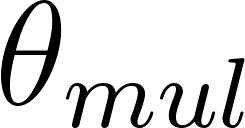](https://www.codecogs.com/eqnedit.php?latex=%5Ctheta_%7Bmul%7D#0) | (3·94–14·45) | Varying | No | No | [39] |
| Relative reduction in  [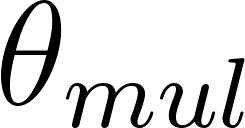](https://www.codecogs.com/eqnedit.php?latex=%5Ctheta_%7Bmul%7D#0) for HIV and ART compartments | - | [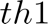](https://www.codecogs.com/eqnedit.php?latex=th1#0) | HIV_0_ = 0  HIV_1_ = 1·00  ART = 0·35 | Fixed | No | No | [8] |
| Relative mortality rate adjustment for HIV and ART compartments | - | [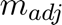](https://www.codecogs.com/eqnedit.php?latex=m_%7Badj%7D#0) | HIV_0_ = 1·00  HIV_1_ = 1·50  ART = 1·15 | Fixed | No | No | [8,22,40,41] |

# Exhibit B. The CHEERS 2022 checklist.

**From: Consolidated Health Economic Evaluation Reporting Standards 2022 (CHEERS 2022) statement: updated reporting guidance for health economic evaluations [42].**

| **Section/topic** | **Item No** | **Guidance for reporting** | **Reported in section** |
| --- | --- | --- | --- |
| **Title** | | | |
| Title | 1 | Identify the study as an economic evaluation and specify the interventions being compared. | Title Page, Paragraph 1 |
| **Abstract** | | | |
| Abstract | 2 | Provide a structured summary that highlights context, key methods, results, and alternative analyses. | Abstract, Paragraphs 1–3 |
| **Introduction** | | | |
| Background and objectives | 3 | Give the context for the study, the study question, and its practical relevance for decision making in policy or practice. | Background, Paragraphs 1–4 |
| **Methods** | | | |
| Health economic analysis plan | 4 | Indicate whether a health economic analysis plan was developed and where available. | Methods, Analytic approach, Paragraph 1 |
| Study population | 5 | Describe characteristics of the study population (such as age range, demographics, socioeconomic, or clinical characteristics). | Methods, Analytic scenarios, Paragraph 1  Exhibit A, section S1.5, Paragraph 1 |
| Setting and location | 6 | Provide relevant contextual information that may influence findings. | Methods, Analytic approach, Paragraph 1  Exhibit C |
| Comparators | 7 | Describe the interventions or strategies being compared and why chosen. | Methods, Analytic approach, Paragraph 1  Methods, Analytic scenarios, Paragraph 1 |
| Perspective | 8 | State the perspective(s) adopted by the study and why chosen. | Methods, Macroeconomic model, Paragraph 1 |
| Time horizon | 9 | State the time horizon for the study and why appropriate. | Methods, Analytic scenarios, Paragraph 1  Exhibit A, section S1.5, Paragraph 1 |
| Discount rate | 10 | Report the discount rate(s) and reason chosen. | Not applicable |
| Selection of outcomes | 11 | Describe what outcomes were used as the measure(s) of benefit(s) and harm(s). | Methods, Outcomes, Paragraph 1 |
| Measurement of outcomes | 12 | Describe how outcomes used to capture benefit(s) and harm(s) were measured. | Methods, Analytic approach, Paragraph 1  Methods, Analytic scenarios, Paragraph 3 |
| Valuation of outcomes | 13 | Describe the population and methods used to measure and value outcomes. | Methods, Analytic approach, Paragraph 1  Methods, Analytic scenarios, Paragraph 3  Methods, Macroeconomic model, Paragraph 1 |
| Measurement and valuation of resources and costs | 14 | Describe how costs were valued. | Methods, Analytic scenarios, Paragraphs 2–3 |
| Currency, price date, and conversion | 15 | Report the dates of the estimated resource quantities and unit costs, plus the currency and year of conversion. | Methods, Analytic scenarios, Paragraph 2 |
| Rationale and description of model | 16 | If modelling is used, describe in detail and why used. Report if the model is publicly available and where it can be accessed. | Exhibit A [1] |
| Analytics and assumptions | 17 | Describe any methods for analyzing or statistically transforming data, any extrapolation methods, and approaches for validating any model used. | Methods, Analytic scenarios, Paragraphs 1–3  Methods, Macroeconomic model, Paragraph 1 |
| Characterizing heterogeneity | 18 | Describe any methods used for estimating how the results of the study vary for subgroups. | Not applicable |
| Characterizing distributional effects | 19 | Describe how impacts are distributed across different individuals or adjustments made to reflect priority populations. | Not applicable |
| Characterizing uncertainty | 20 | Describe methods to characterize any sources of uncertainty in the analysis. | Methods, Statistical analysis, Paragraph 1  Methods, Sensitivity analysis, Paragraphs 1–5 |
| Approach to engagement with patients and others affected by the study | 21 | Describe any approaches to engage patients or service recipients, the general public, communities, or stakeholders (such as clinicians or payers) in the design of the study. | Not applicable |
| **Results** | | | |
| Study parameters | 22 | Report all analytic inputs (such as values, ranges, references) including uncertainty or distributional assumptions. | Exhibit A [1]  Exhibit B  Exhibit C  Exhibit D |
| Summary of main results | 23 | Report the mean values for the main categories of costs and outcomes of interest and summarize them in the most appropriate overall measure. | Results, Total economic impact 2028–2080, Paragraphs 1–2  Results, Time trends in economic impact, Paragraphs 1–3 |
| Effect of uncertainty | 24 | Describe how uncertainty about analytic judgments, inputs, or projections affect findings. Report the effect of choice of discount rate and time horizon, if applicable. | Results, Sensitivity analysis, Paragraphs 1–4 |
| Effect of engagement with patients and others affected by the study | 25 | Report on any difference patient/service recipient, general public, community, or stakeholder involvement made to the approach or findings of the study | Not applicable |
| **Discussion** | | | |
| Study findings, limitations, generalizability, and current knowledge | 26 | Report key findings, limitations, ethical or equity considerations not captured, and how these could affect patients, policy, or practice. | Discussion, Paragraphs 1–8 |
| **Other relevant information** | | | |
| Source of funding | 27 | Describe how the study was funded and any role of the funder in the identification, design, conduct, and reporting of the analysis | Funding statement |
| Conflicts of interest | 28 | Report authors conflicts of interest according to journal or International Committee of Medical Journal Editors requirements. | Competing interests statement |

# Exhibit C. International donor share of TB and HIV/AIDS spending based on development assistance for health spending estimates.

| **Country** | **WHO Region** | **Income level^a^** | **Vaccine introduction year** | **TB international donor share [5]** | **HIV/AIDS international donor share [43]** |
| --- | --- | --- | --- | --- | --- |
| Afghanistan | EMR | LIC | 2031 | 0.79 | 0.79 |
| Angola | AFR | LMIC | 2032 | 0.11 | 0.24 |
| Albania | EUR | UMIC | 2035 | 0.00 | 0.02 |
| Argentina | AMR | UMIC | 2031 | 0.00 | 0.00 |
| Armenia | EUR | UMIC | 2033 | 0.25 | 0.49 |
| Azerbaijan | EUR | UMIC | 2028 | 0.21 | 0.34 |
| Burundi | AFR | LIC | 2044 | 0.45 | 0.94 |
| Benin | AFR | LMIC | 2037 | 0.71 | 0.74 |
| Burkina Faso | AFR | LIC | 2039 | 0.73 | 0.45 |
| Bangladesh | SEAR | LMIC | 2035 | 0.74 | 0.48 |
| Bulgaria | EUR | UMIC | 2029 | 0.05 | 0.04 |
| Belarus | EUR | UMIC | 2028 | 0.03 | 0.21 |
| Bolivia | AMR | LMIC | 2037 | 0.28 | 0.17 |
| Brazil | AMR | UMIC | 2030 | 0.00 | 0.00 |
| Bhutan | SEAR | LMIC | 2034 | 0.28 | 0.29 |
| Botswana | AFR | UMIC | 2028 | 0.36 | 0.24 |
| Central African Republic | AFR | LIC | 2033 | 0.51 | 0.90 |
| China | WPR | UMIC | 2029 | 0.01 | 0.00 |
| Côte d'Ivoire | AFR | LMIC | 2034 | 0.43 | 0.87 |
| Cameroon | AFR | LMIC | 2031 | 0.56 | 0.70 |
| Colombia | AMR | UMIC | 2030 | 0.01 | 0.02 |
| Costa Rica | AMR | UMIC | 2033 | 0.00 | 0.04 |
| Cuba | AMR | UMIC | 2035 | 0.00 | 0.06 |
| Dominican Republic | AMR | UMIC | 2031 | 0.09 | 0.36 |
| Ecuador | AMR | UMIC | 2033 | 0.00 | 0.08 |
| Egypt | EMR | LMIC | 2033 | 0.07 | 0.01 |
| Eritrea | AFR | LIC | 2047 | 0.94 | 0.87 |
| Ethiopia | AFR | LIC | 2030 | 0.47 | 0.87 |
| Fiji | WPR | UMIC | 2031 | 0.20 | 0.01 |
| Gabon | AFR | UMIC | 2038 | 0.28 | 0.04 |
| Georgia | EUR | UMIC | 2029 | 0.48 | 0.38 |
| Ghana | AFR | LMIC | 2040 | 0.59 | 0.51 |
| Guinea | AFR | LIC | 2033 | 0.71 | 0.54 |
| Gambia | AFR | LIC | 2039 | 0.52 | 0.88 |
| Equatorial Guinea | AFR | UMIC | 2042 | 0.03 | 0.01 |
| Guatemala | AMR | UMIC | 2036 | 0.23 | 0.21 |
| Honduras | AMR | LMIC | 2037 | 0.33 | 0.18 |
| Indonesia | SEAR | UMIC | 2034 | 0.36 | 0.32 |
| India | SEAR | LMIC | 2033 | 0.24 | 0.15 |
| Iran | EMR | UMIC | 2031 | 0.06 | 0.03 |
| Iraq | EMR | UMIC | 2033 | 0.07 | 0.01 |
| Jordan | EMR | UMIC | 2037 | 0.15 | 0.35 |
| Kazakhstan | EUR | UMIC | 2028 | 0.06 | 0.14 |
| Kenya | AFR | LMIC | 2032 | 0.39 | 0.50 |
| Kyrgyz Republic | EUR | LMIC | 2044 | 0.31 | 0.59 |
| Cambodia | WPR | LMIC | 2036 | 0.29 | 0.76 |
| Lao People’s Democratic Republic | WPR | LMIC | 2035 | 0.49 | 0.71 |
| Liberia | AFR | LIC | 2037 | 0.98 | 0.58 |
| Libya | EMR | UMIC | 2035 | 0.00 | 0.01 |
| Sri Lanka | SEAR | LMIC | 2028 | 0.15 | 0.15 |
| Lesotho | AFR | LMIC | 2039 | 0.66 | 0.62 |
| Morocco | EMR | LMIC | 2029 | 0.05 | 0.28 |
| Moldova, Republic of | EUR | LMIC | 2034 | 0.14 | 0.59 |
| Madagascar | AFR | LIC | 2031 | 0.62 | 0.69 |
| Maldives | SEAR | UMIC | 2034 | 0.00 | 0.01 |
| Mexico | AMR | UMIC | 2029 | 0.00 | 0.00 |
| Mali | AFR | LIC | 2037 | 0.46 | 0.77 |
| Myanmar | SEAR | LMIC | 2031 | 0.88 | 0.77 |
| Montenegro | EUR | UMIC | 2044 | 0.05 | 0.00 |
| Mongolia | WPR | LMIC | 2032 | 0.15 | 0.44 |
| Mozambique | AFR | LIC | 2032 | 0.72 | 0.92 |
| Mauritania | AFR | LMIC | 2042 | 0.77 | 0.10 |
| Malawi | AFR | LIC | 2038 | 0.91 | 0.85 |
| Malaysia | WPR | UMIC | 2028 | 0.00 | 0.02 |
| Namibia | AFR | UMIC | 2030 | 0.14 | 0.31 |
| Niger | AFR | LIC | 2036 | 0.82 | 0.68 |
| Nigeria | AFR | LMIC | 2030 | 0.63 | 0.63 |
| Nicaragua | AMR | LMIC | 2047 | 0.25 | 0.40 |
| Nepal | SEAR | LMIC | 2036 | 0.35 | 0.31 |
| Pakistan | EMR | LMIC | 2031 | 0.80 | 0.60 |
| Peru | AMR | UMIC | 2029 | 0.03 | 0.01 |
| Philippines | WPR | LMIC | 2030 | 0.36 | 0.13 |
| Papua New Guinea | WPR | LMIC | 2032 | 0.56 | 0.58 |
| Paraguay | AMR | UMIC | 2035 | 0.17 | 0.14 |
| Russian Federation | EUR | UMIC | 2030 | 0.00 | 0.02 |
| Rwanda | AFR | LIC | 2045 | 0.78 | 0.82 |
| Sudan | EMR | LIC | 2036 | 0.87 | 0.68 |
| Senegal | AFR | LMIC | 2038 | 0.39 | 0.74 |
| Solomon Islands | WPR | LMIC | 2047 | 0.44 | 0.07 |
| Sierra Leone | AFR | LIC | 2037 | 0.22 | 0.97 |
| El Salvador | AMR | LMIC | 2039 | 0.29 | 0.15 |
| Serbia | EUR | UMIC | 2036 | 0.00 | 0.01 |
| South Sudan | AFR | LIC | 2034 | 1.00 | 0.98 |
| São Tomé and Principe | AFR | LMIC | 2044 | 0.66 | 0.76 |
| Suriname | AMR | UMIC | 2040 | 0.36 | 0.20 |
| Swaziland | AFR | LMIC | 2036 | 0.79 | 0.62 |
| Syrian Arab Republic | EMR | LIC | 2036 | 0.99 | 0.45 |
| Chad | AFR | LIC | 2033 | 0.48 | 0.68 |
| Togo | AFR | LIC | 2041 | 0.55 | 0.69 |
| Thailand | SEAR | UMIC | 2031 | 0.14 | 0.03 |
| Tajikistan | EUR | LIC | 2045 | 0.36 | 0.68 |
| Timor-Leste | SEAR | LMIC | 2031 | 0.77 | 0.42 |
| Tunisia | EMR | LMIC | 2036 | 0.00 | 0.24 |
| Turkey | EUR | UMIC | 2030 | 0.00 | 0.00 |
| Tanzania, United Republic of | AFR | LMIC | 2031 | 0.69 | 0.89 |
| Uganda | AFR | LIC | 2034 | 0.87 | 0.83 |
| Ukraine | EUR | LMIC | 2033 | 0.24 | 0.38 |
| Uzbekistan | EUR | LMIC | 2038 | 0.36 | 0.47 |
| Venezuela | AMR | UMIC | 2035 | 0.30 | 0.00 |
| Vietnam | WPR | LMIC | 2038 | 0.39 | 0.44 |
| Vanuatu | WPR | LMIC | 2042 | 0.45 | 0.16 |
| Yemen | EMR | LIC | 2036 | 0.87 | 0.34 |
| South Africa | AFR | UMIC | 2029 | 0.12 | 0.20 |
| Zambia | AFR | LIC | 2034 | 0.55 | 0.86 |
| Zimbabwe | AFR | LMIC | 2032 | 0.83 | 0.70 |

^a^ LIC: Gross national income (GNI) per capita of $1,085 or less; LMIC: GNI per capita of $1,086 to $4,225; UMIC: GNI per capita of $4,256 to $13,205 (World Bank 2021).

Note: All countries include 105 low- and middle-income countries analyzed. AFR = African region; AMR = Region of the Americas; EMR = Eastern Mediterranean region; EUR = European region; LIC = low-income country; LMIC = lower middle-income country; SEAR = Southeast Asian region; TB = tuberculosis; UMIC = upper middle-income country; WPR = Western Pacific region.

# Exhibit D. Unit cost inputs and assumptions (2020 USD).

| **Cost item** | **Unit volume** | **Unit costs** | **Source** |
| --- | --- | --- | --- |
| Vaccine price per dose* | Doses delivered | 4.60 | Assumed equal to Gavi-eligible HPV vaccine price [44,45] |
| Supply price per dose* | Doses delivered | 0.11 | [44,45] |
| Infant vaccine delivery costs | Doses delivered | 1.90 (0.56, 5.04) | [46] |
| Adolescent/adult vaccine delivery costs | Doses delivered | 3.50 (1.44, 7.79) | Unpublished; methodology based on [46] |
| One-time introduction costs for infant vaccine in first year of vaccination program** | Age 1 cohort in first year of vaccination program | 0.65 | [47] |
| One-time introduction costs for adolescent/adult vaccine in first year of vaccination program** | Age 9 cohort in first year of vaccination program | 2.40 | [47] |

* Not assumed to vary by country.

** Introduction costs included non-recurrent introduction activity costs to the health system: planning, training, social mobilization, and information, education, communication (IEC).

Note: Values in parentheses represent equal-tailed 95% credible intervals. HPV = human papillomavirus.

# Exhibit E. Technical specifications of the macroeconomic model

The WHO EPIC (Economic Projections of Illness and Cost) model [48-53] is based on a standard human capital augmented Solow growth model, with a linear-homogeneous Cobb-Douglas production function:

| $Y_{it}=\gamma_{i}A_{it}*K_{it}^{\alpha_{it}}*(H_{it}*L_{it})^{1-\alpha_{it}}$ where $0<\alpha<1$, | (1) |
| --- | --- |

where $Y_{it}$ is the GDP of country $i$ in year $t$, $K$ is physical capital, $A_{it}$ is total factor productivity, $L_{it}$ is the aggregate labor force (additional details below), and $H_{it}$ is an educational capital index, i.e., returns to education that increase labor quality. The product $H_{it}*L_{it}$ therefore represents the stock of aggregate labor augmented by education. Total factor productivity $A_{it}$ (which here is Hicks-neutral) enters multiplicatively in the production function as an increasing scale factor. Total factor productivity is an estimate of the change in economic growth that occurs due to factors other than changes in the labor force or capital stock (e.g., technological advancement). The elasticity of output with respect to physical capital is denoted by $\alpha_{it}\in(0, 1)$ and the elasticity of output with respect to labor is denoted by ${(1-\alpha}_{it})\in(0, 1)$. The scaling factor $\gamma_{i}$ is a calibration parameter used to fit the model to reported GDP values (indicator *NY.GDP.MKTP.K* from the World Bank’s World Development Indicators) so that the two are identical in the first year of the projection. For most countries, the initial year used for calibration was 2020. Exceptions, when 2020 data on GDP were not available, were: ERI (2011), SSD (2015) and SYR (2018).

The law of motion for physical capital is given by the equation:

| $K_{it}=s_{it}Y_{i(t-1)}+(1-\delta_{it})K_{i(t-1)}$, | (2) |
| --- | --- |

where $s_{it}$ is the savings rate, which was assumed to be exogenous, as well as country- and year-specific. The depreciation rate $\delta_{it}$ was also assumed to be country- and year-specific.

We extracted estimates for the savings rate $s_{it}$ from the World Bank’s World Development Indicators (indicator: *NE.GDI.TOTL.ZS*) [54]. We extracted estimates for the depreciation rate $\delta_{it}$ from the latest Penn World Tables (PWT; indicator: *delta*) [55]. Under the assumption of competitive markets, the elasticity of output with respect to physical capital $\alpha_{it}$ is equal to labor income as a share of GDP. We derived values for this variable from ILOSTAT (indicator: *LAP_2GDP_NOC_RT_A*) [56].

The labor force was defined as the stock of individuals of working age (ages 15–69) who participate in the labor market, with data organized by single-year age cohorts. Historical estimates (up to 2020) and future projections (to 2080) of demographic inputs were obtained from the UN Population Division (World Population Prospects 2019 [9]), with $N_{ait}$ denoting the population of age group $a$ in country $i$ in year $t$.

The raw stock of labor for each age group was calculated by multiplying the base population by the labor force participation rate $p_{ai}$ for the relevant age group using the latest available country-specific data (indicator *EAP_DWAP_SEX_AGE_RT_A* from the ILOSTAT library) [56]. These data are available in five-year age intervals: 15–19, 20–24, 25–29, 30–34, 35–39, 40–44, 45–49, 50–54, 55–59, 60–64, ≥65. The size of the labor force in each age group is therefore calculated as $L_{ait}=p_{ai}N_{ait}$.

These age-specific stocks of labor were then augmented by experience using a standard Cuddington and Hancock (1994) specification [57]. Skill-augmented labor $L_{it}$ is defined as:

| $L_{it}=\sum_{a=15}^{60} \rho_{a}L_{ait},$ | (3) |
| --- | --- |

where $\rho_{a}$ is the Cuddington/skill-augmenting factor that adjusts for skill and experience for different ages and was defined as:

| $\rho_{a}=\rho_{1}+\rho_{2}\left( {age}_{a}-15 \right)+\rho_{3}({age}_{a}-15)^{2},$ | (4) |
| --- | --- |

where ${age}_{a}$ is the age of a worker while $\rho_{1}$ = 0.8, $\rho_{2}$ = 0.02, and $\rho_{3}$ = -0.0002.

Educational capital and total factor productivity were assumed to grow at rates $h_{it}$ and $g_{it}$, respectively:

| $H_{it}=H_{i(t-1)}e^{h_{it}}, A_{it}=A_{i(t-1)}e^{g_{it}}.$ | (5) |
| --- | --- |

The growth rates of educational attainment and total factor productivity were computed from the levels reported in the PWT [55,58,59]. The specific PWT variables from which we computed the annual growth rates were *rtfpna* (for $g_{it}$) and *hc* (for $h_{it}$). The educational attainment index *hc* was based on years of schooling [58] and returns to education [59]. The construction of total factor productivity *rtfpna* in the PWT is detailed in [55].

When costs are introduced, the evolution of physical capital can be rewritten as:

| $K_{it}=s_{it}Y_{i(t-1)}-C_{it}+(1-\delta_{it})K_{i(t-1)}$ , | (6) |
| --- | --- |

where $C_{it}$ is the public sector cost of TB vaccination (net of international donor contributions), net of patient out-of-pocket and public expenditure treatment savings.

Improved health status stemming from the intervention has positive effects on the size of the effective labor force due to reduced mortality, as established by the *Mtb* transmission model. This counterfactual labor force is constructed by replacing the projected $N_{ait}$ series estimated by the transmission model with the simulation projection $\tilde{N}_{ait}$ and proceeding as detailed above.

The increase in the projected labor force by $\tilde{L}_{it}-L_{it}$ is then the main source of gains in GDP from the novel TB vaccine intervention, which is mitigated when net intervention costs $C_{it}$ are strictly positive.

The initial level of the stock of physical capital per efficiency unit of labor is calibrated so as to correspond to its steady-state level $k_{it}^{*}$ given by:

| $k_{it}^{*}=(\frac{s_{it}\gamma_{i}A_{it}}{n_{it}+h_{it}+\delta_{it}})^{\frac{1}{1-\alpha_{it}}} ,$ | (7) |
| --- | --- |

where $k\equiv K/HL$. This avoids unrealistically high growth rates generated by what would correspond to transitional dynamics toward the steady-state at the start of the simulation, and therefore yields the balanced growth path.

# Exhibit F. Cumulative absolute gains to gross domestic product (GDP, billions US$2020) due to adolescent/adult tuberculosis vaccines by decade.

| Country grouping | 2041–2050 | 2051–2060 | 2061–2070 | 2071–2080 |
| --- | --- | --- | --- | --- |
| All countries | 111 (43.5, 201) | 283 (126, 528) | 482 (204, 955) | 729 (284, 1518) |
| High-TB burden^a^ | 113 (54.0, 197) | 280 (135, 511) | 471 (212, 920) | 708 (290, 1457) |
| High-TB/HIV burden^a^ | 105 (54.9, 176) | 254 (127, 453) | 420 (194, 804) | 621 (262, 1250) |
| High-MDR/RR-TB burden^a^ | 109 (52.6, 190) | 269 (130, 491) | 451 (203, 883) | 678 (277, 1397) |
|  | Income level^b^ | | | |
| LIC | 2.58 (0.61, 5.19) | 8.30 (2.88, 16.3) | 15.9 (5.32, 32.7) | 26.5 (8.24, 56.7) |
| LMIC | 97.2 (49.6, 165) | 248 (122, 449) | 423 (191, 824) | 642 (262, 1320) |
| UMIC | 11.2 (-6.70, 30.2) | 27.0 (1.34, 62.2) | 42.5 (7.26, 97.9) | 60.0 (13.2, 141) |
|  | World region | | | |
| AFR | 36.7 (19.8, 60.6) | 77.6 (40.0, 135) | 114 (54.1, 212) | 151 (66.1, 296) |
| AMR | -2.07 (-5.34, -0.37) | -1.54 (-4.83, 0.44) | -1.16 (-4.43, 1.07) | -0.90 (-4.17, 1.51) |
| EMR | -1.17 (-3.65, 0.96) | 1.33 (-2.31, 5.32) | 3.39 (-1.56, 9.51) | 5.27 (-1.02, 13.5) |
| EUR | -0.64 (-2.97, 0.96) | 0.11 (-2.86, 2.80) | 0.86 (-3.06, 5.13) | 1.95 (-3.19, 8.53) |
| SEAR | 64.7 (33.9, 110) | 169 (83.2, 308) | 293 (133, 573) | 448 (184, 915) |
| WPR | 13.4 (1.74, 29.1) | 36.8 (12.9, 76.3) | 71.4 (26.0, 155) | 123 (42.3, 284) |

Note: Values in parentheses represent equal-tailed 95% uncertainty intervals.

^a^ High-TB, high-TB/HIV (HIV-associated TB), and high-MDR/RR-TB (multidrug/rifampicin-resistant TB) burden countries as defined by the World Health Organization.

^b^ LIC: Gross national income (GNI) per capita of $1,085 or less; LMIC: GNI per capita of $1,086 to $4,225; UMIC: GNI per capita of $4,256 to $13,205 (World Bank 2021).

Note: All countries include 105 low- and middle-income countries analyzed. AFR = African region; AMR = Region of the Americas; EMR = Eastern Mediterranean region; EUR = European region; GDP = gross domestic product; LIC = low-income; LMIC = lower middle-income; SEAR = Southeast Asian region; UMIC = upper middle-income; WPR = Western Pacific region.

# Exhibit G. Cumulative absolute gains to gross domestic product (GDP, billions US$2020) due to infant tuberculosis vaccines by decade.

| Country grouping | 2041–2050 | 2051–2060 | 2061–2070 | 2071–2080 |
| --- | --- | --- | --- | --- |
| All countries | -0.75 (-5.17, 3.62) | 18.4 (0.75, 44.1) | 62.0 (16.1, 138) | 128 (36.1, 288) |
| High-TB burden^a^ | 0.98 (-2.10, 4.60) | 21.6 (6.97, 45.3) | 65.9 (25, 138) | 132 (47.6, 286) |
| High-TB/HIV burden^a^ | 1.51 (-0.86, 4.59) | 19.3 (6.86, 39.6) | 57.3 (22.4, 118) | 112 (41.7, 239) |
| High-MDR/RR-TB burden^a^ | 1.10 (-1.88, 4.62) | 20.9 (6.71, 43.9) | 63.5 (23.9, 133) | 127 (45.6, 275) |
|  | Income level^b^ | | | |
| LIC | -0.26 (-0.46, -0.09) | -0.003 (-0.80, 0.93) | 0.96 (-0.96, 3.59) | 2.79 (-1.03, 8.47) |
| LMIC | 0.97 (-1.47, 4.06) | 17.9 (5.01, 38.3) | 55.6 (19.5, 119) | 113 (38.4, 247) |
| UMIC | -1.46 (-3.24, -0.35) | 0.55 (-3.46, 4.82) | 5.43 (-2.47, 16.0) | 12.2 (-1.21, 32.7) |
|  | World region | | | |
| AFR | 0.66 (-0.67, 2.42) | 7.67 (1.77, 16.9) | 19.4 (6.11, 41.6) | 32.7 (10.0, 70.5) |
| AMR | -0.52 (-0.92, -0.30) | -0.83 (-1.56, -0.44) | -1.02 (-2.01, -0.50) | -1.16 (-2.35, -0.51) |
| EMR | -0.67 (-1.11, -0.37) | -0.71 (-1.77, 0.18) | -0.50 (-2.22, 1.17) | -0.17 (-2.62, 2.39) |
| EUR | -0.41 (-0.80, -0.22) | -0.70 (-1.46, -0.32) | -0.84 (-1.97, -0.22) | -0.88 (-2.43, 0.12) |
| SEAR | 0.52 (-0.38, 1.66) | 9.27 (3.40, 19.0) | 31.3 (12.3, 65.1) | 66.2 (24.5, 142) |
| WPR | -0.33 (-1.28, 0.42) | 3.70 (0.36, 8.73) | 13.7 (3.90, 30.9) | 31.0 (9.05, 73.8) |

Note: Values in parentheses represent equal-tailed 95% uncertainty intervals.

^a^ High-TB, high-TB/HIV (HIV-associated TB), and high-MDR/RR-TB (multidrug/rifampicin-resistant TB) burden countries as defined by the World Health Organization.

^b^ LIC: Gross national income (GNI) per capita of $1,085 or less; LMIC: GNI per capita of $1,086 to $4,225; UMIC: GNI per capita of $4,256 to $13,205 (World Bank 2021).

Note: All countries include 105 low- and middle-income countries analyzed. AFR = African region; AMR = Region of the Americas; EMR = Eastern Mediterranean region; EUR = European region; GDP = gross domestic product; LIC = low-income; LMIC = lower middle-income; SEAR = Southeast Asian region; UMIC = upper middle-income; WPR = Western Pacific region.

# Exhibit H. Gains to gross domestic product (GDP) due to infant tuberculosis (TB) vaccines across 2028–2080 for 105 analyzed low- and middle-income countries by vaccine characteristic and delivery scenario.

| Scenario | Absolute gains in GDP  (billions US$2020) | Percentage gain in GDP  (%) |
| --- | --- | --- |
| All countries | 207 (80.6, 405) | 0.0041% (0.0029%, 0.0053%) |
| Lifelong duration of protection | 429 (170, 886) | 0.0082% (0.0062%, 0.0101%) |
| Low coverage | 199 (72.4, 427) | 0.0038% (0.0027%, 0.0048%) |
| High coverage | 249 (89.7, 534) | 0.0047% (0.0033%, 0.006%) |
| Accelerated scale-up | 765 (314, 1526) | 0.0146% (0.0112%, 0.0183%) |
| Rapid TB decline | 24.7 (-22.1, 109) | 0.0004% (-0.0007%, 0.0014%) |

Note: Values in parentheses represent equal-tailed 95% uncertainty intervals.

#

# Exhibit I. Gains to gross domestic product (GDP) due to adolescent/adult tuberculosis vaccines across 2028–2080: health services costs included government-level and excluded patient- and international-donor-level.

| Country grouping | Absolute gains in GDP  (billions US$2020) | Percentage gain in GDP  (%) |
| --- | --- | --- |
| All countries | 1552 (715, 2900) | 0.0311% (0.0254%, 0.0372%) |
| High-TB burden^a^ | 1528 (716, 2833) | 0.0380% (0.0316%, 0.0452%) |
| High-TB/HIV burden^a^ | 1366 (653, 2498) | 0.0823% (0.0693%, 0.0974%) |
| High-MDR/RR-TB burden^a^ | 1469 (690, 2710) | 0.0368% (0.0305%, 0.0439%) |
|  | Income level^b^ | |
| LIC | 43.0 (16.3, 90.7) | 0.0308% (0.0213%, 0.0400%) |
| LMIC | 1371 (637, 2531) | 0.0810% (0.0690%, 0.0955%) |
| UMIC | 138 (35.5, 294) | 0.0043% (0.0021%, 0.0058%) |
|  | World region | |
| AFR | 367 (189, 654) | 0.1105% (0.0942%, 0.1279%) |
| AMR | -6.29 (-18.2, -0.45) | -0.0021% (-0.0058%, -0.0001%) |
| EMR | -1.37 (-9.74, 8.59) | -0.0016% (-0.0103%, 0.0046%) |
| EUR | 0.95 (-9.28, 11.4) | 0.00004% (-0.0028%, 0.0015%) |
| SEAR | 957 (439, 1785) | 0.0823% (0.0667%, 0.1003%) |
| WPR | 235 (86.6, 495) | 0.0089% (0.0064%, 0.0115%) |

Note: Values in parentheses represent equal-tailed 95% uncertainty intervals.

^a^ High-TB, high-TB/HIV (HIV-associated TB), and high-MDR/RR-TB (multidrug/rifampicin-resistant TB) burden countries as defined by the World Health Organization.

^b^ LIC: Gross national income (GNI) per capita of $1,085 or less; LMIC: GNI per capita of $1,086 to $4,225; UMIC: GNI per capita of $4,256 to $13,205 (World Bank 2021).

Note: All countries include 105 low- and middle-income countries analyzed. AFR = African region; AMR = Region of the Americas; EMR = Eastern Mediterranean region; EUR = European region; GDP = gross domestic product; LIC = low-income; LMIC = lower middle-income; SEAR = Southeast Asian region; UMIC = upper middle-income; WPR = Western Pacific region.

# Exhibit J. Gains to gross domestic product (GDP) due to infant tuberculosis vaccines across 2028–2080: health services costs included government-level and excluded patient- and international-donor-level.

| Country grouping | Absolute gains in GDP  (billions US$2020) | Percentage gain in GDP  (%) |
| --- | --- | --- |
| All countries | 198 (73.0, 395) | 0.0039% (0.0026%, 0.0052%) |
| High-TB burden^a^ | 211 (85.1, 406) | 0.0052% (0.0038%, 0.0066%) |
| High-TB/HIV burden^a^ | 184 (78.3, 345) | 0.0110% (0.0079%, 0.0143%) |
| High-MDR/RR-TB burden^a^ | 204 (82.7, 393) | 0.0051% (0.0037%, 0.0064%) |
| Income level^b^ | Income level^b^ | |
| LIC | 1.98 (-1.37, 8.05) | 0.0011% (-0.0017%, 0.0035%) |
| LMIC | 180 (72.7, 349) | 0.0105% (0.0075%, 0.0138%) |
| UMIC | 16.4 (-0.94, 43.7) | 0.0005% (-0.00004%, 0.0008%) |
| World region | World region | |
| AFR | 58.4 (24.1, 116) | 0.0174% (0.0114%, 0.0243%) |
| AMR | -3.35 (-5.82, -2.17) | -0.0011% (-0.0019%, -0.0006%) |
| EMR | -4.35 (-7.05, -2.21) | -0.0034% (-0.0064%, -0.0012%) |
| EUR | -2.74 (-5.5, -1.46) | -0.0007% (-0.0016%, -0.0003%) |
| SEAR | 104 (42.2, 207) | 0.0089% (0.0060%, 0.0125%) |
| WPR | 46.0 (14.6, 102) | 0.0017% (0.0010%, 0.0025%) |

Note: Values in parentheses represent equal-tailed 95% uncertainty intervals.

^a^ High-TB, high-TB/HIV (HIV-associated TB), and high-MDR/RR-TB (multidrug/rifampicin-resistant TB) burden countries as defined by the World Health Organization.

^b^ LIC: Gross national income (GNI) per capita of $1,085 or less; LMIC: GNI per capita of $1,086 to $4,225; UMIC: GNI per capita of $4,256 to $13,205 (World Bank 2021).

Note: All countries include 105 low- and middle-income countries analyzed. AFR = African region; AMR = Region of the Americas; EMR = Eastern Mediterranean region; EUR = European region; GDP = gross domestic product; LIC = low-income; LMIC = lower middle-income; SEAR = Southeast Asian region; UMIC = upper middle-income; WPR = Western Pacific region.

# Exhibit K. Gains to gross domestic product (GDP) due to adolescent/adult tuberculosis vaccines across 2028–2080: health services costs included patient-level and excluded government- and international-donor-level.

| Country grouping | Absolute gains in GDP  (billions US$2020) | Percentage gain in GDP  (%) |
| --- | --- | --- |
| All countries | 1772 (875, 3211) | 0.0358% (0.0305%, 0.0421%) |
| High-TB burden^a^ | 1685 (835, 3049) | 0.0421% (0.0358%, 0.0494%) |
| High-TB/HIV burden^a^ | 1470 (741, 2649) | 0.0890% (0.0765%, 0.1035%) |
| High-MDR/RR-TB burden^a^ | 1615 (799, 2911) | 0.0407% (0.0345%, 0.0478%) |
|  | Income level^b^ | |
| LIC | 67.9 (35.3, 123) | 0.0503% (0.0435%, 0.0577%) |
| LMIC | 1498 (741, 2713) | 0.0889% (0.0771%, 0.1030%) |
| UMIC | 206 (97.1, 374) | 0.0066% (0.0056%, 0.0076%) |
|  | World region | |
| AFR | 418 (228, 728) | 0.1268% (0.1110%, 0.1441%) |
| AMR | 8.67 (4.39, 15.0) | 0.0027% (0.0021%, 0.0032%) |
| EMR | 26.9 (15.6, 44.1) | 0.0197% (0.0149%, 0.0256%) |
| EUR | 15.0 (7.38, 27.1) | 0.0035% (0.0029%, 0.0041%) |
| SEAR | 1015 (484, 1862) | 0.0876% (0.0719%, 0.1052%) |
| WPR | 288 (127, 551) | 0.0111% (0.0092%, 0.0133%) |

Note: Values in parentheses represent equal-tailed 95% uncertainty intervals.

^a^ High-TB, high-TB/HIV (HIV-associated TB), and high-MDR/RR-TB (multidrug/rifampicin-resistant TB) burden countries as defined by the World Health Organization.

^b^ LIC: Gross national income (GNI) per capita of $1,085 or less; LMIC: GNI per capita of $1,086 to $4,225; UMIC: GNI per capita of $4,256 to $13,205 (World Bank 2021).

Note: All countries include 105 low- and middle-income countries analyzed. AFR = African region; AMR = Region of the Americas; EMR = Eastern Mediterranean region; EUR = European region; GDP = gross domestic product; LIC = low-income; LMIC = lower middle-income; SEAR = Southeast Asian region; UMIC = upper middle-income; WPR = Western Pacific region.

# Exhibit L. Gains to gross domestic product (GDP) due to infant tuberculosis vaccines across 2028–2080: health services costs included patient-level and excluded government- and international-donor-level.

| Country grouping | Absolute gains in GDP  (billions US$2020) | Percentage gain in GDP  (%) |
| --- | --- | --- |
| All countries | 257 (120, 470) | 0.0052% (0.0041%, 0.0064%) |
| High-TB burden^a^ | 249 (116, 456) | 0.0062% (0.0050%, 0.0077%) |
| High-TB/HIV burden^a^ | 211 (99.8, 380) | 0.0127% (0.0100%, 0.0160%) |
| High-MDR/RR-TB burden^a^ | 239 (111, 438) | 0.0060% (0.0048%, 0.0075%) |
| Income level^b^ | Income level^b^ | |
| LIC | 9.39 (4.74, 17.1) | 0.0069% (0.0057%, 0.0083%) |
| LMIC | 214 (99.5, 395) | 0.0127% (0.0101%, 0.0158%) |
| UMIC | 33.1 (14.2, 63.5) | 0.0010% (0.0008%, 0.0013%) |
| World region | World region | |
| AFR | 76.5 (38.6, 140) | 0.0231% (0.0178%, 0.0299%) |
| AMR | 0.53 (0.17, 1.06) | 0.0002% (0.0001%, 0.0002%) |
| EMR | 5.33 (2.94, 8.98) | 0.0039% (0.0027%, 0.0056%) |
| EUR | 1.07 (0.38, 2.35) | 0.0002% (0.0002%, 0.0003%) |
| SEAR | 116 (51.9, 220) | 0.0100% (0.0073%, 0.0135%) |
| WPR | 57.3 (23.6, 116) | 0.0022% (0.0016%, 0.0029%) |

Note: Values in parentheses represent equal-tailed 95% uncertainty intervals.

^a^ High-TB, high-TB/HIV (HIV-associated TB), and high-MDR/RR-TB (multidrug/rifampicin-resistant TB) burden countries as defined by the World Health Organization.

^b^ LIC: Gross national income (GNI) per capita of $1,085 or less; LMIC: GNI per capita of $1,086 to $4,225; UMIC: GNI per capita of $4,256 to $13,205 (World Bank 2021).

Note: All countries include 105 low- and middle-income countries analyzed. AFR = African region; AMR = Region of the Americas; EMR = Eastern Mediterranean region; EUR = European region; GDP = gross domestic product; LIC = low-income; LMIC = lower middle-income; SEAR = Southeast Asian region; UMIC = upper middle-income; WPR = Western Pacific region.

# Exhibit M. Gains to gross domestic product (GDP) due to adolescent/adult tuberculosis vaccines across 2028–2080: health services costs included all levels (government, patient, international donor).

| Country grouping | Absolute gains in GDP  (billions US$2020) | Percentage gain in GDP  (%) |
| --- | --- | --- |
| All countries | 1523 (698, 2849) | 0.0306% (0.0247%, 0.0367%) |
| High-TB burden^a^ | 1536 (720, 2833) | 0.0382% (0.0314%, 0.0455%) |
| High-TB/HIV burden^a^ | 1368 (654, 2491) | 0.0825% (0.0694%, 0.0977%) |
| High-MDR/RR-TB burden^a^ | 1483 (698, 2715) | 0.0372% (0.0305%, 0.0445%) |
|  | Income level^b^ | |
| LIC | 11.8 (-16.1, 50.1) | 0.0067% (-0.0188%, 0.0227%) |
| LMIC | 1369 (642, 2516) | 0.0809% (0.0681%, 0.0954%) |
| UMIC | 142.9 (38.6, 303) | 0.0044% (0.0022%, 0.0060%) |
|  | World region | |
| AFR | 337 (167, 607) | 0.1011% (0.0801%, 0.1200%) |
| AMR | -7.20 (-20.4, -1.10) | -0.0024% (-0.0065%, -0.0002%) |
| EMR | -7.30 (-21.7, 5.22) | -0.0060% (-0.0194%, 0.0028%) |
| EUR | -0.25 (-11.6, 10.1) | -0.00025% (-0.0033%, 0.0014%) |
| SEAR | 958 (441, 1781) | 0.0824% (0.0665%, 0.1009%) |
| WPR | 244 (92.3, 504) | 0.0093% (0.0066%, 0.0118%) |

Note: Values in parentheses represent equal-tailed 95% uncertainty intervals.

^a^ High-TB, high-TB/HIV (HIV-associated TB), and high-MDR/RR-TB (multidrug/rifampicin-resistant TB) burden countries as defined by the World Health Organization.

^b^ LIC: Gross national income (GNI) per capita of $1,085 or less; LMIC: GNI per capita of $1,086 to $4,225; UMIC: GNI per capita of $4,256 to $13,205 (World Bank 2021).

Note: All countries include 105 low- and middle-income countries analyzed. AFR = African region; AMR = Region of the Americas; EMR = Eastern Mediterranean region; EUR = European region; GDP = gross domestic product; LIC = low-income; LMIC = lower middle-income; SEAR = Southeast Asian region; UMIC = upper middle-income; WPR = Western Pacific region.

# Exhibit N. Gains to gross domestic product (GDP) due to infant tuberculosis vaccines across 2028–2080: health services costs included all levels (government, patient, international donor).

| Country grouping | Absolute gains in GDP  (billions US$2020) | Percentage gain in GDP  (%) |
| --- | --- | --- |
| All countries | 171 (51.1, 370) | 0.0033% (0.0018%, 0.0047%) |
| High-TB burden^a^ | 201 (77.4, 391) | 0.0049% (0.0034%, 0.0064%) |
| High-TB/HIV burden^a^ | 173 (69.2, 334) | 0.0103% (0.0071%, 0.0137%) |
| High-MDR/RR-TB burden^a^ | 199 (78.9, 387) | 0.0049% (0.0035%, 0.0064%) |
| Income level^b^ | Income level^b^ | |
| LIC | -13.1 (-20.4, -8.30) | -0.0106% (-0.0201%, -0.0043%) |
| LMIC | 168 (64.3, 335) | 0.0098% (0.0065%, 0.0132%) |
| UMIC | 16.3 (-1.28, 43.7) | 0.0005% (-0.0001%, 0.0008%) |
| World region | World region | |
| AFR | 55.8 (20.7, 111) | 0.0168% (0.0096%, 0.0242%) |
| AMR | -3.58 (-6.33, -2.27) | -0.0012% (-0.0021%, -0.0007%) |
| EMR | -5.96 (-11.2, -2.23) | -0.0046% (-0.0092%, -0.0014%) |
| EUR | -3.21 (-6.37, -1.74) | -0.0008% (-0.0018%, -0.0003%) |
| SEAR | 105 (42.5, 208) | 0.0089% (0.0060%, 0.0126%) |
| WPR | 49.7 (17.0, 107) | 0.0019% (0.0012%, 0.0026%) |

Note: Values in parentheses represent equal-tailed 95% uncertainty intervals.

^a^ High-TB, high-TB/HIV (HIV-associated TB), and high-MDR/RR-TB (multidrug/rifampicin-resistant TB) burden countries as defined by the World Health Organization.

^b^ LIC: Gross national income (GNI) per capita of $1,085 or less; LMIC: GNI per capita of $1,086 to $4,225; UMIC: GNI per capita of $4,256 to $13,205 (World Bank 2021).

Note: All countries include 105 low- and middle-income countries analyzed. AFR = African region; AMR = Region of the Americas; EMR = Eastern Mediterranean region; EUR = European region; GDP = gross domestic product; LIC = low-income; LMIC = lower middle-income; SEAR = Southeast Asian region; UMIC = upper middle-income; WPR = Western Pacific region.

# Exhibit O. Gains to gross domestic product (GDP) due to adolescent/adult tuberculosis vaccines across 2028–2080: low-growth scenario.

| Country grouping | Absolute gains in GDP  (billions US$2020) | Percentage gain in GDP  (%) |
| --- | --- | --- |
| All countries | 1060 (812, 1399) | 0.0312% (0.0255%, 0.0385%) |
| High-TB burden^a^ | 1049 (802, 1386) | 0.0383% (0.0304%, 0.0494%) |
| High-TB/HIV burden^a^ | 948 (727, 1245) | 0.0816% (0.0688%, 0.0962%) |
| High-MDR/RR-TB burden^a^ | 1009 (766, 1342) | 0.0372% (0.0293%, 0.0475%) |
|  | Income level^b^ | |
| LIC | 32.6 (24.2, 41.0) | 0.0371% (0.0307%, 0.0443%) |
| LMIC | 941 (698, 1263) | 0.0815% (0.0690%, 0.0954%) |
| UMIC | 87.0 (44.8, 138) | 0.0040% (0.0022%, 0.0054%) |
|  | World region | |
| AFR | 269 (194, 356) | 0.1171% (0.0975%, 0.1419%) |
| AMR | -6.40 (-12.4, -3.20) | -0.0026% (-0.0053%, -0.0012%) |
| EMR | 4.00 (-2.13, 10.4) | 0.0041% (-0.0024%, 0.0107%) |
| EUR | -0.50 (-5.79, 3.21) | -0.0002% (-0.0021%, 0.0011%) |
| SEAR | 648 (413, 994) | 0.0810% (0.0645%, 0.0996%) |
| WPR | 146 (89.5, 203) | 0.0085% (0.0061%, 0.0120%) |

Note: Values in parentheses represent equal-tailed 95% uncertainty intervals.

^a^ High-TB, high-TB/HIV (HIV-associated TB), and high-MDR/RR-TB (multidrug/rifampicin-resistant TB) burden countries as defined by the World Health Organization.

^b^ LIC: Gross national income (GNI) per capita of $1,085 or less; LMIC: GNI per capita of $1,086 to $4,225; UMIC: GNI per capita of $4,256 to $13,205 (World Bank 2021).

Note: All countries include 105 low- and middle-income countries analyzed. AFR = African region; AMR = Region of the Americas; EMR = Eastern Mediterranean region; EUR = European region; GDP = gross domestic product; LIC = low-income; LMIC = lower middle-income; SEAR = Southeast Asian region; UMIC = upper middle-income; WPR = Western Pacific region.

# Exhibit P. Gains to gross domestic product (GDP) due to infant tuberculosis vaccines across 2028–2080: low-growth scenario.

| Country grouping | Absolute gains in GDP  (billions US$2020) | Percentage gain in GDP  (%) |
| --- | --- | --- |
| All countries | 125 (82.6, 178) | 0.0037% (0.0026%, 0.0049%) |
| High-TB burden^a^ | 137 (95.4, 188) | 0.0050% (0.0037%, 0.0068%) |
| High-TB/HIV burden^a^ | 122 (82.8, 170) | 0.0105% (0.0080%, 0.0135%) |
| High-MDR/RR-TB burden^a^ | 133 (90.8, 184) | 0.0049% (0.0036%, 0.0067%) |
| Income level^b^ | Income level^b^ | |
| LIC | 1.19 (-0.18, 2.62) | 0.0013% (-0.0002%, 0.0027%) |
| LMIC | 118 (78.4, 167) | 0.0102% (0.0077%, 0.0132%) |
| UMIC | 6.72 (-1.74, 16.2) | 0.0003% (-0.0001%, 0.0006%) |
| World region | World region | |
| AFR | 40.1 (20.8, 66.1) | 0.0174% (0.0103%, 0.0262%) |
| AMR | -3.10 (-4.30, -2.44) | -0.0013% (-0.0018%, -0.0009%) |
| EMR | -2.30 (-4.21, -0.49) | -0.0024% (-0.0044%, -0.0005%) |
| EUR | -2.63 (-4.04, -1.78) | -0.0009% (-0.0016%, -0.0005%) |
| SEAR | 67.6 (37.9, 109) | 0.0084% (0.0058%, 0.0123%) |
| WPR | 25.7 (13.9, 38.6) | 0.0015% (0.0009%, 0.0021%) |

Note: Values in parentheses represent equal-tailed 95% uncertainty intervals.

^a^ High-TB, high-TB/HIV (HIV-associated TB), and high-MDR/RR-TB (multidrug/rifampicin-resistant TB) burden countries as defined by the World Health Organization.

^b^ LIC: Gross national income (GNI) per capita of $1,085 or less; LMIC: GNI per capita of $1,086 to $4,225; UMIC: GNI per capita of $4,256 to $13,205 (World Bank 2021).

Note: All countries include 105 low- and middle-income countries analyzed. AFR = African region; AMR = Region of the Americas; EMR = Eastern Mediterranean region; EUR = European region; GDP = gross domestic product; LIC = low-income; LMIC = lower middle-income; SEAR = Southeast Asian region; UMIC = upper middle-income; WPR = Western Pacific region.

# Exhibit Q. Gains to gross domestic product (GDP) due to adolescent/adult tuberculosis vaccines across 2028–2080: high-growth scenario.

| Country grouping | Absolute gains in GDP  (billions US$2020) | Percentage gain in GDP  (%) |
| --- | --- | --- |
| All countries | 2295 (1796, 3036) | 0.0345% (0.0249%, 0.0457%) |
| High-TB burden^a^ | 2239 (1740, 2974) | 0.0418% (0.0283%, 0.0560%) |
| High-TB/HIV burden^a^ | 1972 (1489, 2712) | 0.0897% (0.0779%, 0.1064%) |
| High-MDR/RR-TB burden^a^ | 2142 (1653, 2831) | 0.0404% (0.0273%, 0.0542%) |
|  | Income level^b^ | |
| LIC | 80.3 (59.9, 114) | 0.0412% (0.0338%, 0.0493%) |
| LMIC | 2004 (1523, 2686) | 0.0872% (0.0750%, 0.1021%) |
| UMIC | 211 (128, 371) | 0.0050% (0.0038%, 0.0063%) |
|  | World region | |
| AFR | 525 (404, 715) | 0.1163% (0.0991%, 0.1406%) |
| AMR | -6.46 (-13.8, -1.49) | -0.0016% (-0.0036%, -0.0003%) |
| EMR | 13.8 (3.23, 27.2) | 0.0074% (0.0017%, 0.0138%) |
| EUR | 4.81 (-3.20, 15.7) | 0.0008% (-0.0006%, 0.0022%) |
| SEAR | 1383 (978, 1947) | 0.0884% (0.0721%, 0.1077%) |
| WPR | 375 (259, 593) | 0.0108% (0.0079%, 0.0156%) |

Note: Values in parentheses represent equal-tailed 95% uncertainty intervals.

^a^ High-TB, high-TB/HIV (HIV-associated TB), and high-MDR/RR-TB (multidrug/rifampicin-resistant TB) burden countries as defined by the World Health Organization.

^b^ LIC: Gross national income (GNI) per capita of $1,085 or less; LMIC: GNI per capita of $1,086 to $4,225; UMIC: GNI per capita of $4,256 to $13,205 (World Bank 2021).

Note: All countries include 105 low- and middle-income countries analyzed. AFR = African region; AMR = Region of the Americas; EMR = Eastern Mediterranean region; EUR = European region; GDP = gross domestic product; LIC = low-income; LMIC = lower middle-income; SEAR = Southeast Asian region; UMIC = upper middle-income; WPR = Western Pacific region.

# Exhibit R. Gains to gross domestic product (GDP) due to infant tuberculosis vaccines across 2028–2080: high-growth scenario.

| Country grouping | Absolute gains in GDP  (billions US$2020) | Percentage gain in GDP  (%) |
| --- | --- | --- |
| All countries | 306 (223, 440) | 0.0046% (0.0032%, 0.0063%) |
| High-TB burden^a^ | 320 (239, 455) | 0.0060% (0.0040%, 0.0085%) |
| High-TB/HIV burden^a^ | 273 (189, 407) | 0.0124% (0.0099%, 0.0162%) |
| High-MDR/RR-TB burden^a^ | 308 (225, 434) | 0.0058% (0.0038%, 0.0084%) |
| Income level^b^ | Income level^b^ | |
| LIC | 6.55 (2.88, 11.9) | 0.0033% (0.0017%, 0.0050%) |
| LMIC | 272 (187, 395) | 0.0119% (0.0092%, 0.0156%) |
| UMIC | 27.6 (12.2, 57.5) | 0.0006% (0.0004%, 0.0009%) |
| World region | World region | |
| AFR | 85.2 (55.9, 143) | 0.0188% (0.0132%, 0.0285%) |
| AMR | -4.32 (-6.10, -3.17) | -0.0011% (-0.0016%, -0.0007%) |
| EMR | -1.87 (-5.42, 1.39) | -0.001% (-0.0029%, 0.0008%) |
| EUR | -3.36 (-6.35, -1.73) | -0.0006% (-0.0013%, -0.0003%) |
| SEAR | 155 (96.2, 231) | 0.0099% (0.0070%, 0.0138%) |
| WPR | 75.6 (45.9, 128) | 0.0022% (0.0015%, 0.0031%) |

Note: Values in parentheses represent equal-tailed 95% uncertainty intervals.

^a^ High-TB, high-TB/HIV (HIV-associated TB), and high-MDR/RR-TB (multidrug/rifampicin-resistant TB) burden countries as defined by the World Health Organization.

^b^ LIC: Gross national income (GNI) per capita of $1,085 or less; LMIC: GNI per capita of $1,086 to $4,225; UMIC: GNI per capita of $4,256 to $13,205 (World Bank 2021).

Note: All countries include 105 low- and middle-income countries analyzed. AFR = African region; AMR = Region of the Americas; EMR = Eastern Mediterranean region; EUR = European region; GDP = gross domestic product; LIC = low-income; LMIC = lower middle-income; SEAR = Southeast Asian region; UMIC = upper middle-income; WPR = Western Pacific region.

# Exhibit S. Gains to gross domestic product (GDP) due to adolescent/adult tuberculosis vaccines across 2028–2080: scenario with alternative approach to modelling the consequences of TB morbidity outcomes.

| Country grouping | Absolute gains in GDP  (billions US$2020) | Percentage gain in GDP  (%) |
| --- | --- | --- |
| All countries | 1338 (654, 2422) | 0.0270% (0.0221%, 0.0322%) |
| High-TB burden^a^ | 1316 (654, 2364) | 0.0329% (0.0273%, 0.0390%) |
| High-TB/HIV burden^a^ | 1199 (606, 2113) | 0.0726% (0.0618%, 0.0839%) |
| High-MDR/RR-TB burden^a^ | 1256 (623, 2247) | 0.0317% (0.0262%, 0.0377%) |
|  | Income level^b^ | |
| LIC | 51.0 (26.2, 89.6) | 0.0378% (0.0306%, 0.0451%) |
| LMIC | 1183 (583, 2111) | 0.0702% (0.0602%, 0.0809%) |
| UMIC | 103 (20.9, 222) | 0.0032% (0.0009%, 0.0045%) |
|  | World region | |
| AFR | 345 (189, 590) | 0.1047% (0.0901%, 0.1197%) |
| AMR | -7.43 (-20.2, -1.78) | -0.0024% (-0.0064%, -0.0005%) |
| EMR | 9.91 (-0.01, 21.8) | 0.0071% (-0.00001%, 0.013%) |
| EUR | -1.21 (-11.4, 6.28) | -0.0004% (-0.0031%, 0.0010%) |
| SEAR | 813 (387, 1474) | 0.0701% (0.0571%, 0.0837%) |
| WPR | 178 (70.7, 364) | 0.0068% (0.0049%, 0.0086%) |

Note: Values in parentheses represent equal-tailed 95% uncertainty intervals.

^a^ High-TB, high-TB/HIV (HIV-associated TB), and high-MDR/RR-TB (multidrug/rifampicin-resistant TB) burden countries as defined by the World Health Organization.

^b^ LIC: Gross national income (GNI) per capita of $1,085 or less; LMIC: GNI per capita of $1,086 to $4,225; UMIC: GNI per capita of $4,256 to $13,205 (World Bank 2021).

Note: All countries include 105 low- and middle-income countries analyzed. AFR = African region; AMR = Region of the Americas; EMR = Eastern Mediterranean region; EUR = European region; GDP = gross domestic product; LIC = low-income; LMIC = lower middle-income; SEAR = Southeast Asian region; UMIC = upper middle-income; WPR = Western Pacific region.

# Exhibit T. Gains to gross domestic product (GDP) due to infant tuberculosis vaccines across 2028–2080: scenario with alternative approach to modelling the consequences of TB morbidity outcomes.

| Country grouping | Absolute gains in GDP  (billions US$2020) | Percentage gain in GDP  (%) |
| --- | --- | --- |
| All countries | 187 (73.6, 364) | 0.0037% (0.0026%, 0.0048%) |
| High-TB burden^a^ | 198 (84.8, 370) | 0.0049% (0.0037%, 0.0062%) |
| High-TB/HIV burden^a^ | 173 (76.7, 316) | 0.0104% (0.0078%, 0.0131%) |
| High-MDR/RR-TB burden^a^ | 191 (81.8, 353) | 0.0048% (0.0035%, 0.006%) |
| Income level^b^ | Income level^b^ | |
| LIC | 4.38 (0.82, 10.2) | 0.0031% (0.0009%, 0.0047%) |
| LMIC | 168 (72.1, 316) | 0.0099% (0.0073%, 0.0126%) |
| UMIC | 14.5 (-2.00, 38.1) | 0.0004% (-0.0001%, 0.0008%) |
| World region | World region | |
| AFR | 55.1 (23.9, 107) | 0.0165% (0.0114%, 0.0227%) |
| AMR | -3.53 (-6.22, -2.25) | -0.0011% (-0.0021%, -0.0007%) |
| EMR | -1.08 (-4.10, 1.58) | -0.0009% (-0.0035%, 0.0011%) |
| EUR | -2.97 (-6.02, -1.58) | -0.0008% (-0.0017%, -0.0003%) |
| SEAR | 96.0 (40.0, 186) | 0.0082% (0.0057%, 0.0114%) |
| WPR | 43.0 (14.6, 91.9) | 0.0016% (0.0010%, 0.0023%) |

Note: Values in parentheses represent equal-tailed 95% uncertainty intervals.

^a^ High-TB, high-TB/HIV (HIV-associated TB), and high-MDR/RR-TB (multidrug/rifampicin-resistant TB) burden countries as defined by the World Health Organization.

^b^ LIC: Gross national income (GNI) per capita of $1,085 or less; LMIC: GNI per capita of $1,086 to $4,225; UMIC: GNI per capita of $4,256 to $13,205 (World Bank 2021).

Note: All countries include 105 low- and middle-income countries analyzed. AFR = African region; AMR = Region of the Americas; EMR = Eastern Mediterranean region; EUR = European region; GDP = gross domestic product; LIC = low-income; LMIC = lower middle-income; SEAR = Southeast Asian region; UMIC = upper middle-income; WPR = Western Pacific region.

# References

1. Clark RA, Mukandavire C, Portnoy A, Weerasuriya CK, Deol A, Scarponi D, et al. The impact of alternative delivery strategies for novel tuberculosis vaccines in low- and middle-income countries: a modelling study. Lancet Glob Health. 2023;11(4):e546-e55.

2. Frascella B, Richards AS, Sossen B, Emery JC, Odone A, Law I, et al. Subclinical Tuberculosis Disease-A Review and Analysis of Prevalence Surveys to Inform Definitions, Burden, Associations, and Screening Methodology. Clin Infect Dis. 2021;73(3):e830-e41.

3. Emery JC, Richards AS, Dale KD, McQuaid CF, White RG, Denholm JT, et al. Self-clearance of Mycobacterium tuberculosis infection: implications for lifetime risk and population at-risk of tuberculosis disease. Proc Biol Sci. 2021;288(1943):20201635.

4. World Health Organization. Guidelines for treatment of drug-susceptible tuberculosis and patient care: 2017 update. 2017. World Health Organization. https://apps.who.int/iris/handle/10665/255052. License: CC BY-NC-SA 3.0 IGO.

5. World Health Organization. Global Tuberculosis Report 2022. Geneva: World Health Organization. 27 October 2022. [Online] Accessed 27 October 2022. Available at: https://www.who.int/teams/global-tuberculosis-programme/data.

6. Kwan CK, Ernst JD. HIV and tuberculosis: a deadly human syndemic. Clin Microbiol Rev. 2011;24(2):351-76.

7. Vynnycky E, Fine PE. The natural history of tuberculosis: the implications of age-dependent risks of disease and the role of reinfection. Epidemiol Infect. 1997;119(2):183-201.

8. Suthar AB, Lawn SD, del Amo J, Getahun H, Dye C, Sculier D, et al. Antiretroviral therapy for prevention of tuberculosis in adults with HIV: a systematic review and meta-analysis. PLoS Med. 2012;9(7):e1001270.

9. United Nations. World Population Prospects. United Nations Population Division. https://population.un.org/wpp/ (accessed 18 Jul 2022). 2020.

10. World Health Organization. WHO Global TB Programme (2022). WHO Global TB Database [retrieved from https://www.who.int/teams/global-tuberculosis-programme/data, Nov 23 2022]. Geneva, Switzerland, WHO Global TB Programme.

11. Iskauskas A. hmer: History Matching and Emulation Package. R package. 17 May 2022. Available at: https://CRAN.R-project.org/package=hmer (accessed 12 July 2022).

12. Scarponi D, Iskauskas A, Clark RA, Vernon I, McKinley TJ, Goldstein M, et al. Demonstrating Multi-Country Calibration of a Tuberculosis Model Using New History Matching and Emulation Package - Hmer. medRxiv; 2022. doi: https://doi.org/10.1101/2022.05.13.22275052.

13. Jabot F, Faure T, Dumoulin N, Albert C. EasyABC: Efficient Approximate Bayesian Computation Sampling Schemes. R package. 2015. Available at: https://CRAN.R-project.org/package=EasyABC (accessed April 2022).

14. World Health Organization. WHO Preferred Product Characteristics for New Tuberculosis Vaccines. Geneva: World Health Organization. Licence: CC BY-NC-SA 3.0 IGO. 13 July 2018. https://www.who.int/publications/i/item/WHO-IVB-18.06 (accessed 18 October 2021).

15. Abubakar I, Pimpin L, Ariti C, Beynon R, Mangtani P, Sterne JA, et al. Systematic review and meta-analysis of the current evidence on the duration of protection by bacillus Calmette-Guérin vaccination against tuberculosis. Health Technol Assess. 2013;17(37):1-372, v-vi.

16. Blackwood JC, Cummings DA, Broutin H, Iamsirithaworn S, Rohani P. Deciphering the impacts of vaccination and immunity on pertussis epidemiology in Thailand. Proc Natl Acad Sci U S A. 2013;110(23):9595-600.

17. Lewnard JA, Grad YH. Vaccine waning and mumps re-emergence in the United States. Sci Transl Med. 2018;10(433).

18. Gavi The Vaccine Alliance. Country hub. Geneva: Gavi, The Vaccine Alliance. https://www.gavi.org/programmes-impact/country-hub (accessed 15 April 2022).

19. UNICEF. Vaccination and Immunization Statistics. https://data.unicef.org/topic/child-health/immunization/ (accessed March 7, 2022). .

20. Harris RC, Sumner T, Knight GM, Zhang H, White RG. Potential impact of tuberculosis vaccines in China, South Africa, and India. Sci Transl Med. 2020;12(564).

21. Fu H, Lewnard JA, Frost I, Laxminarayan R, Arinaminpathy N. Modelling the global burden of drug-resistant tuberculosis avertable by a post-exposure vaccine. Nat Commun. 2021;12(1):424.

22. Tiemersma EW, van der Werf MJ, Borgdorff MW, Williams BG, Nagelkerke NJ. Natural history of tuberculosis: duration and fatality of untreated pulmonary tuberculosis in HIV negative patients: a systematic review. PLoS One. 2011;6(4):e17601.

23. Quaife M, Houben R, Allwood B, Cohen T, Coussens AK, Harries AD, et al. Post-tuberculosis mortality and morbidity: valuing the hidden epidemic. Lancet Respir Med. 2020;8(4):332-3.

24. Silva S, Awad S, Abu-Raddad L, Atun R, Goosby E, Reid M. The Health and Economic Benefits Possible with Novel Tuberculosis Vaccines – A Modeling Study in India and Indonesia. rs.3.rs-265017v1. [preprint] 2021 [cited 9 January 2023]. Available from: https://doi.org/10.21203/rs.3.rs-265017/v1.

25. Smith RD, Keogh-Brown MR, Barnett T, Tait J. The economy-wide impact of pandemic influenza on the UK: a compuTable A.5eneral equilibrium modelling experiment. BMJ. 2009;339:b4571.

26. Smith RD, Yago M, Millar M, Coast J. Assessing the macroeconomic impact of a healthcare problem: the application of compuTable A.5eneral equilibrium analysis to antimicrobial resistance. J Health Econ. 2005;24(6):1055-75.

27. Abu-Raddad LJ, Sabatelli L, Achterberg JT, Sugimoto JD, Longini IM, Jr., Dye C, et al. Epidemiological benefits of more-effective tuberculosis vaccines, drugs, and diagnostics. Proc Natl Acad Sci U S A. 2009;106(33):13980-5.

28. Dye C, Williams BG. Eliminating human tuberculosis in the twenty-first century. J R Soc Interface. 2008;5(23):653-62.

29. Marx FM, Dunbar R, Enarson DA, Williams BG, Warren RM, van der Spuy GD, et al. The temporal dynamics of relapse and reinfection tuberculosis after successful treatment: a retrospective cohort study. Clin Infect Dis. 2014;58(12):1676-83.

30. Gomes GM, Franco AO, Gomes MC, Medley GF. The reinfection threshold promotes variability in tuberculosis epidemiology and vaccine efficacy. Proc Biol Sci. 2004;271(1539):617-23.

31. Dangisso MH, Woldesemayat EM, Datiko DG, Lindtjørn B. Long-term outcome of smear-positive tuberculosis patients after initiation and completion of treatment: A ten-year retrospective cohort study. PLoS One. 2018;13(3):e0193396.

32. World Health Organization. Treatment Outcomes. CSV Files to Download. 2022. (accessed Nov 2, 2022). Available at: https://www.who.int/tb/country/data/download/en/

33. Sutherland I, Svandová E, Radhakrishna S. The development of clinical tuberculosis following infection with tubercle bacilli. 1. A theoretical model for the development of clinical tuberculosis following infection, linking from data on the risk of tuberculous infection and the incidence of clinical tuberculosis in the Netherlands. Tubercle. 1982;63(4):255-68.

34. Gomes GM, Rodrigues P, Hilker FM, Mantilla-Beniers NB, Muehlen M, Cristina Paulo A, et al. Implications of partial immunity on the prospects for tuberculosis control by post-exposure interventions. J Theor Biol. 2007;248(4):608-17.

35. Mberi MN, Kuonza LR, Dube NM, Nattey C, Manda S, Summers R. Determinants of loss to follow-up in patients on antiretroviral treatment, South Africa, 2004-2012: a cohort study. BMC Health Serv Res. 2015;15:259.

36. Van Cutsem G, Ford N, Hildebrand K, Goemaere E, Mathee S, Abrahams M, et al. Correcting for mortality among patients lost to follow up on antiretroviral therapy in South Africa: a cohort analysis. PLoS One. 2011;6(2):e14684.

37. Johansson KA, Robberstad B, Norheim OF. Further benefits by early start of HIV treatment in low income countries: survival estimates of early versus deferred antiretroviral therapy. AIDS Res Ther. 2010;7(1):3.

38. Fatti G, Mothibi E, Meintjes G, Grimwood A. Antiretroviral treatment outcomes amongst older adults in a large multicentre cohort in South Africa. PLoS One. 2014;9(6):e100273.

39. Vazquez F. A systematic review and meta-analysis of the effect of HIV status on the incidence of tuberculosis disease among individuals with latent Mycobacterium tuberculosis infection. [Masters Dissertation]. 2019.

40. Ackah AN, Coulibaly D, Digbeu H, Diallo K, Vetter KM, Coulibaly IM, et al. Response to treatment, mortality, and CD4 lymphocyte counts in HIV-infected persons with tuberculosis in Abidjan, Côte d'Ivoire. Lancet. 1995;345(8950):607-10.

41. Mukadi YD, Maher D, Harries A. Tuberculosis case fatality rates in high HIV prevalence populations in sub-Saharan Africa. Aids. 2001;15(2):143-52.

42. Husereau D, Drummond M, Augustovski F, de Bekker-Grob E, Briggs AH, Carswell C, et al. Consolidated Health Economic Evaluation Reporting Standards 2022 (CHEERS 2022) Statement: Updated Reporting Guidance for Health Economic Evaluations. BMJ. 2022;376:e067975.

43. Global Burden of Disease Collaborative Network. Global HIV/AIDS Spending 2000-2017. Seattle, United States of America: Institute for Health Metrics and Evaluation (IHME), 2020. https://doi.org/10.6069/0PTH-YJ74 (accessed 28 July 2022).

44. UNICEF. Vaccines pricing data. 8 March 2022. https://www.unicef.org/supply/vaccines-pricing-data (accessed 17 March 2022).

45. UNICEF. Costs of Vaccinating a Child. 14 August 2020. https://immunizationeconomics.org/recent-activity/2021/6/15/standard-costs-of-vaccinating-a-child (accessed 20 January 2021).

46. Portnoy A, Vaughan K, Clarke-Deelder E, Suharlim C, Resch SC, Brenzel L, et al. Producing Standardized Country-Level Immunization Delivery Unit Cost Estimates. Pharmacoeconomics. 2020;38(9):995-1005.

47. Gavi The Vaccine Alliance. GAVI Alliance Vaccine Introduction Grant and Operational Support for Campaigns Policy. Version No. 1.0. Geneva: Gavi, The Vaccine Alliance. 2 April 2013. www.gavi.org (accessed 12 July 2018).

48. Abegunde D, Stanciole A. An estimation of the economic impact of chronic noncommunicable diseases in selected countries. 2006:1-21.

49. Alkire BC, Peters AW, Shrime MG, Meara JG. The Economic Consequences Of Mortality Amenable To High-Quality Health Care In Low- And Middle-Income Countries. Health Aff (Millwood). 2018;37(6):988-96.

50. Beaulieu N, Bloom DE, Bloom LR, Stein R. Breakaway: The global burden of cancer challenges and opportunities. The Economist Intelligence Unit, London: Livestrong, 2009.

51. Bloom DE, Cafiero ET, Jané-Llopis E, Abrahams-Gessel S, Bloom LR, Fathima S, et al. The Global Economic Burden of Non-communicable Diseases. Geneva: World Economic Forum, 2011. https://www.weforum.org/reports/global-economic-burden-non-communicable-diseases/ (accessed 2 December 2020).

52. Bloom DE, Cafiero-Fonseca ET, McGovern ME, Prettner K, Stanciole A, Weiss J, et al. The macroeconomic impact of non-communicable diseases in China and India: Estimates, projections, and comparisons. J Econ Ageing. 2014;4:100-11.

53. Bloom DE, Chen S, Kuhn M, McGovern ME, Oxley L, Prettner K. The economic burden of chronic diseases: estimates and projections for China, Japan and South Korea, NBER working paper 23601, 2017. https://www.nber.org/papers/w23601 (accessed 2 December 2020).

54. World Bank. World development indicators. Washington, DC: The World Bank; 2021. http://data.worldbank.org/ (accessed 18 July 2022).

55. Feenstra R, Inklaar R, Timmer M. The Next Generation of the Penn Word Table. Am Econ Rev. 2015;105(10):3150--82.

56. International Labour Organization. “Employment by sex and age — ILO modelled estimates.” ILOSTAT database, https://ilostat.ilo.org/data/ (accessed 18 July 2022).

57. Cuddington JT, Hancock JD. Assessing the impact of AIDS on the growth path of the Malawian economy. J Dev Econ. 1994;43(2):363-8.

58. Barro RJ, Lee JW. A new data set of educational attainment in the world, 1950–2010. J Dev Econ. 2013;104:184-98.

59. Psacharopoulos G. Returns to investment in education: A global update. World Dev. 1994;22(9):1325-43.
